# Quantum nondemolition measurement of mechanical motion quanta

**DOI:** 10.1038/s41467-018-06070-y

**Published:** 2018-09-06

**Authors:** Luca Dellantonio, Oleksandr Kyriienko, Florian Marquardt, Anders S. Sørensen

**Affiliations:** 1 The Niels Bohr Institute, University of Copenhagen, Blegdamsvej 17, 2100 Copenhagen Ø, Denmark; 2 Center for Hybrid Quantum Networks (Hy-Q), The Niels Bohr Institute, University of Copenhagen, Blegdamsvej 17, 2100 Copenhagen Ø, Denmark; 3 NORDITA, KTH Royal Institute of Technology and Stockholm University, Roslagstullsbacken 23, 106 91 Stockholm, Sweden; 4 Institute for Theoretical Physics, University Erlangen-Nürnberg, Staudstraße 7, 91058 Erlangen, Germany; 5 Max Planck Institute for the Science of Light, Günther-Scharowsky-Straße 1, 91058 Erlangen, Germany

## Abstract

The fields of optomechanics and electromechanics have facilitated numerous advances in the areas of precision measurement and sensing, ultimately driving the studies of mechanical systems into the quantum regime. To date, however, the quantization of the mechanical motion and the associated quantum jumps between phonon states remains elusive. For optomechanical systems, the coupling to the environment was shown to make the detection of the mechanical mode occupation difficult, typically requiring the single-photon strong-coupling regime. Here, we propose and analyse an electromechanical setup, which allows us to overcome this limitation and resolve the energy levels of a mechanical oscillator. We found that the heating of the membrane, caused by the interaction with the environment and unwanted couplings, can be suppressed for carefully designed electromechanical systems. The results suggest that phonon number measurement is within reach for modern electromechanical setups.

## Abstract (web summary)

Although electro-and optomechanics has recently moved towards the quantum regime, the quantized energy spectrum of a mechanical oscillator has not been directly observed. Here Dellantonio et al. propose an electromechanical setup with a membrane resonator that could enable phonon number measurements.

## Introduction

Energy quantization is one of the hallmarks of quantum mechanics. First theorized for light by Einstein and Planck, it was found to be ubiquitous in nature and represents a cornerstone of modern physics. It has been observed in various microscopic systems starting from nuclei, atoms and molecules, to larger mesoscopic condensed matter systems such as superconductors^1^. For macroscopic systems, however, the observation of energy quantization is hindered by the smallness of the Planck constant. Thus, although being a milestone of contemporary physics, up to date the discrete energy spectrum of mechanical resonators has never been seen directly.

Extreme progress in studying mechanical systems has been achieved in experiments exploiting radiation pressure. This is the core of optomechanics^2^, where photons and phonons of the optical and mechanical subsystems interact with each other. A similar type of coupling can be realized in the microwave domain with electrical circuits, leading to the field of electromechanics^3–8^. The numerous advances of optomechanics and electromechanics include ground state cooling^4,5,9–11^, ultra precise sensing^12–15^, generation of squeezed light and mechanical states^7,8,16–18^, back action cancellation^19,20^ and detection of gravitational waves^21^. In all of these systems, however, the operation in the single-photon/phonon regime is challenging due to the small value of the bare coupling^3,22^. Instead, experiments exploit an enhanced linearized effective coupling induced by a large driving field. This severely limits the nature of the interactions^23^ and possible quantum effects. In particular, it precludes the observation of the energy quantization in mechanical resonators.

Quantization of mechanical energy can be observed by a quantum nondemolition (QND) measurement^24,25^ of an oscillator’s phonon number operator $\hat n_{\mathrm{b}}$. Here, QND means that the interaction, which couples the mechanical system with the measurement apparatus, does not affect the observable we are interested in. This is achieved if the total Hamiltonian commutes with $\hat n_{\mathrm{b}}$, and the influence of the environment is minimized.

**Fig. 1:**
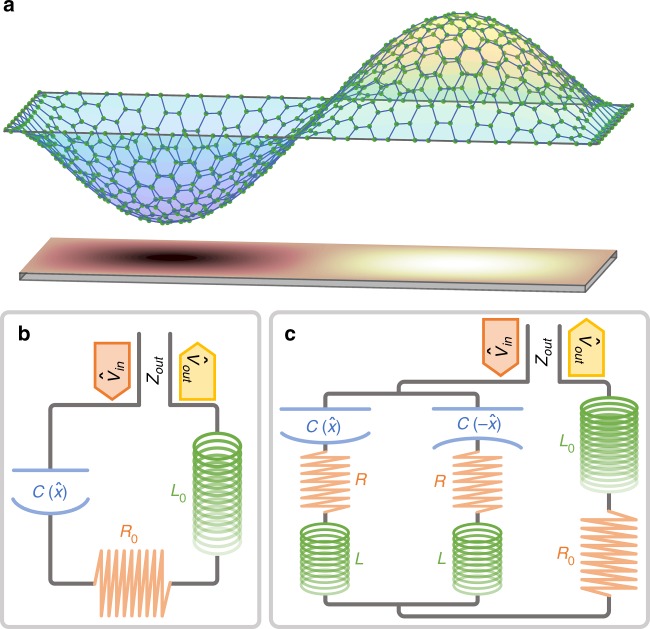
Experimental setup. **a** Sketch of a capacitor with an oscillating plate, here represented by a graphene membrane. We consider an antisymmetric (2, 1) mechanical mode. **b** *RLC* oscillator formed by the inductance *L*_0_, resistance *R*_0_, and position-dependent capacitance $C(\hat x)$. The circuit is driven by the input voltage $\hat V_{{\mathrm{in}}}$ through a transmission line of impedance *Z*_out_. $\hat V_{{\mathrm{out}}}$ is the reflected signal. **c** Model circuit for an *RLC* system where the capacitor has the same form as in **a**. The membrane has a vanishing linear coupling to the symmetric electrical mode used for probing the system. The antisymmetric mode, residing in the small loop containing parasitic inductances *L* and resistances *R*, describes the redistribution of charge on the capacitor

Considering the electromechanical setups in Fig. 1, we show that QND detection is feasible for a capacitor in which one of the electrodes is a light micromechanical oscillator. By choosing an antisymmetric mode for the oscillator, the interaction between the electrical and mechanical subsystems is quadratic in the displacement. Along with the suppression of the linear coupling, this ensures the QND nature of the measurement, as originally proposed in refs. ^26,27^ for an optomechanical system. In that system, however, it was shown in refs. ^28,29^ that the combination of unwanted losses and the coupling to an orthogonal electromagnetic mode spoils the interaction, unless strong single-photon coupling is achieved. Here, we show that for the considered electromechanical setup the equivalent orthogonal mode can have dramatically different properties, allowing for the phonon QND detection. We derive general conditions under which the QND measurement is possible, and characterize its experimental signatures. As compared to most approaches to phonon QND measurement^26,27,30–32^, our procedure does not impose stringent requirements on the single-photon optomechanical coupling, but relies on the ratio of the involved coupling constants. This makes our approach attractive even for systems where the interaction is limited, for example, due to stray capacitances in the setup. For a measurement of the square displacement, a similar advantage was identified in ref.  ^31^.

## Results

### Proceeding

We first study an *RLC* circuit with one capacitor plate being an oscillating membrane, without assuming the symmetry discussed above (Fig. 1b). The mechanical motion of the plate shifts the resonance frequency of the circuit, while the electric potential exerts a force on the membrane. In order to perform a QND measurement of the phonon number, we require this interaction to be proportional to $\hat n_{\mathrm{b}}$. We therefore Taylor expand the inverse of the capacitance to second order in the displacement, $1{\mathrm{/}}C(\hat x)$ ≃ $C_0^{ - 1}$ + $\tilde g_1(\hat b + \hat b^\dagger )$ + $\tilde g_2( {\hat b + \hat b^\dagger } )^2{\mathrm{/}}2$, where we replaced the position $\hat x$ with the creation $\hat b^\dagger$ and annihilation $\hat b$ operators of the mechanical motion, and $\tilde g_{1,2}$ denote linear and quadratic coupling constants. Within the rotating wave approximation, $\tilde g_2 ( {\hat b + \hat b^\dagger } )^2{\mathrm{/}}2$ ≃ $\tilde g_2\hat n_{\mathrm{b}}$, leading to the desired QND interaction, while the $\tilde g_1$ term adds unwanted heating that spoils the phonon measurement.

**Fig. 2:**
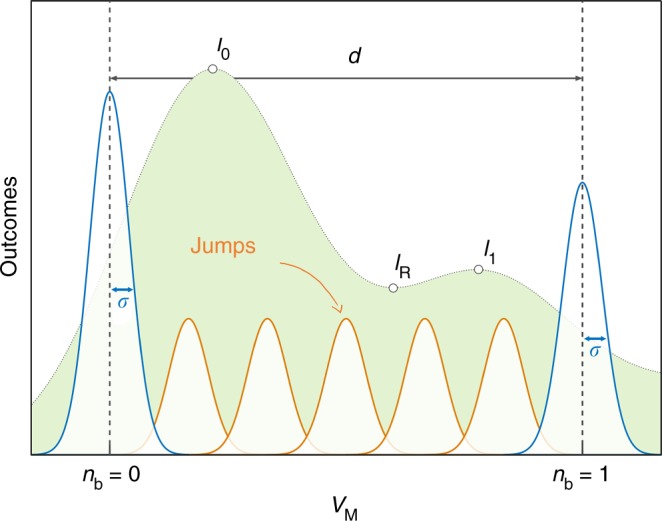
Sketch of the experimental outcome. Distribution of outcomes *V*_M_ for two different phonon numbers: *n*_b_ = 0 (first peak to the left) and *n*_b_ = 1 (last peak to the right). For a given value of *n*_b_, repeated measurements are Gaussian distributed with a variance $\sigma ^2 \propto 1 + 2\bar n_{\mathrm{e}}$ of the outgoing signal $\hat V_{{\mathrm{out}}}$, consisting of vacuum and thermal noise. The distance *d* between the two peaks depends on the circuit parameters and the number of incident photons, and identifies the signal-to-noise ratio *D* = *d*/*σ*. Ideally, for each shot of the measurement, the mechanics is either in its ground or first excited state. However, for Δ*n*_b_ > 0 there will be events where the mechanical state jumps, resulting in outcomes *V*_M_ in between the peaks relative to *n*_b_ = 0 and *n*_b_ = 1 (smaller peaks in the figure). This leads to the smeared distribution shown in the back. The visibility of the QND measurement is quantified by the values at the peaks and valleys, as indicated by *I*_0_, *I*_1_ and *I*_R_ (see Eq. (9)). The figure is for illustration only, and is not to scale

The main aim of this work is to identify conditions under which the QND measurement is feasible, despite the presence of heating. We initially consider the simple circuit in Fig. 1b, and assume the incoming signal $\hat V_{{\mathrm{in}}}$ to be in a coherent state resonant with the circuit. The quadratic interaction then shifts the electrical resonance frequency proportionally to the phonon number $\tilde g_2\hat n_{\mathrm{b}}$. For small $\tilde g_2$, this shift leads to a phase change of the outgoing signal $\hat V_{{\mathrm{out}}}$ that can be determined by homodyne measurement. Different phononic states will thus lead to distinct outcomes *V*_M_, as shown in Fig. 2. The distance *d* between output signals for different $\hat n_{\mathrm{b}}$ and the standard deviation *σ* of the noise define the signal-to-noise ratio *D* = *d*/*σ* (see Fig. 2), which needs to be maximized.

In order to have a successful QND measurement, the phonon number $\hat n_{\mathrm{b}}$ must be conserved. If the mechanical state jumps during a measurement, the outcome *V*_M_ ends up between the desired peaks. This leads to a reduced contrast, as illustrated by the distribution in the background of Fig. 2. The probability for $\hat n_{\mathrm{b}}$ to change is generally state-dependent, in the sense that higher Fock states are more likely to jump. A state-independent characterization of this heating is given by the average phonons Δ*n*_b_ added to the ground state during the measurement time *T*. The jump probability for any state can then be derived from Δ*n*_b_ using standard results for harmonic oscillators (for details see Supplementary Note 3 available in  Supplementary Material online).

Both *D* and Δ*n*_b_ are proportional to the incoming intensity. We therefore characterize a setup by the parameter *λ* = *D*^2^/Δ*n*_b_, where $\lambda \gg 1$ is required for successful QND detection. For the *RLC* circuit in Fig. 1b, we find below that

1 $$\lambda = \frac{1}{{2\left( {1 + 2\bar n_{\mathrm{e}}} \right)^2}}\left( {\frac{{g_2}}{{g_1}}} \right)^2\left( {\frac{{\omega _{\mathrm{m}}}}{{\gamma _{\mathrm{t}}}}} \right)^2,$$

where $g_1 = \tilde g_1C_0\omega _{\mathrm{s}}$, $g_2 = \tilde g_2C_0\omega _{\mathrm{s}}$ and $\bar n_{\mathrm{e}}$ is the thermal occupation of *R*_0_ and *Z*_out_ (assumed equal, *R*_0_ = *Z*_out_). Here, *ω*_m_ and *ω*_s_ = (*C*_0_*L*_0_)^−1/2^ ≫ *ω*_m_ are the mechanical and electrical frequencies, respectively, and *γ*_t_ = *Z*_out_/*L*_0_ corresponds to the output coupling rate. A result similar to Eq. (1) is derived in ref. ^33^.

Despite progresses in reaching the resolved sideband regime $\omega _{\mathrm{m}} \gg \gamma _{\mathrm{t}}$ in both optomechanical and electromechanical systems, *g*_2_ is generally much smaller than *g*_1_, implying $\lambda \ll 1$ in Eq. (1). To circumvent this problem, we use the second fundamental mode of the membrane in the capacitor, as depicted in Fig. 1a. The first-order coefficient $\tilde g_1$ of the $1{\mathrm{/}}C(\hat x)$ expansion then vanishes, leaving $\tilde g_2$ to be the largest contribution to the electromechanical coupling. In this situation *λ* seemingly grows indefinitely, the induced heating disappears and the QND measurement of the phonon number is easily realized. In practice, however, two effects will limit the achievable value of *λ*. First, inaccuracies in the nanofabrication can cause misalignments and, consequently, a residual linear coupling. Second, the oscillation of the membrane induces a charge redistribution in the capacitor to maintain it at an equipotential. The associated antisymmetric electrical mode introduces an effective linear coupling, and a similar heating mechanism as the one identified in ref.^28^ for the optomechanical setup of refs^26,27^. In these papers, the quadratic interaction results from a hybridization of two modes linearly coupled to the mechanical position, and the QND detection was found to be impossible unless the single-photon coupling *g*_1_ exceeded the intrinsic cavity damping. In our case, the QND interaction arises directly from the Taylor expansion of the capacitance. Hence, there is no constraint tying the second-order coupling *g*_2_ to the properties of the symmetric and antisymmetric electrical modes, which can have vastly different resonance frequencies and dampings. This inhibits the mechanical heating and ultimately allows for the QND detection of the phonon number. We model the charge redistribution in the capacitor by parasitic inductances (*L*) and resistances (*R*) in the equivalent circuit of Fig. 1c. Each of the two arms containing *R* and *L* represents one half of the capacitor, with opposite dependence on the membrane position, $C(\hat x)$ and $C( - \hat x)$.

### Single-arm *RLC* circuit

In the following, we derive Eq. (1) for the *RLC* circuit in Fig. 1b. The methods sketched here will then be generalised for the double-arm circuit in Fig. 1c. Using the standard approach^34^, we write the circuit Hamiltonian as $\hat {\cal H}(\hat x)$ = ${\hat{\mathrm \Phi }}^2{\mathrm{/}}\left[ {2L_0} \right] + \hat Q^2{\mathrm{/}}\left[ {2C(\hat x)} \right]$, where the conjugate variables $\hat Q$ and ${\hat{\mathrm \Phi }}$ are the charge and magnetic flux, respectively. We can expand $\hat {\cal H}(\hat x)$ in the mechanical position $\hat x \propto \hat b + \hat b^\dagger$, in order to obtain the circuit Hamiltonian $\hat {\cal H}_{\mathrm{e}}$ = $\hat {\cal H}\left( {\hat x = 0} \right)$ and the coupling Hamiltonian $\hat {\cal H}_{{\mathrm{em}}}$ = $g_1\omega _{\mathrm{s}}L_0\hat Q^2 ( {\hat b + \hat b^\dagger } ){\mathrm{/}}2$ + $g_2\omega _{\mathrm{s}}L_0\hat Q^2 ( {\hat n_{\mathrm{b}} + \hat b\hat b{\mathrm{/}}2 + \hat b^\dagger \hat b^\dagger {\mathrm{/}}2} )$. The total Hamiltonian $\hat {\cal H}_{{\mathrm{tot}}}$ = $\hat {\cal H}_{\mathrm{e}} + \hat {\cal H}_{{\mathrm{em}}} + \hat {\cal H}_{\mathrm{m}}$ is therefore the sum of the circuit, interaction and the mechanical Hamiltonian $\hat {\cal H}_{\mathrm{m}}$ = $\hbar \omega _{\mathrm{m}}\hat b^\dagger \hat b$.

Next, we describe the environmental effects corresponding to decay and heating of the modes. Associating each resistor *R*_i_ with its own Johnson–Nyquist noise $\hat V_{R_{\mathrm{i}}}$, we find the equations of motion of the composite system

2 $$\begin{array}{*{20}{c}} . \\ {\hat Q} \\ {} \end{array} = \frac{{{\hat{\mathrm \Phi }}}}{{L_0}},$$

3 $$\begin{array}{*{20}{l}} {\begin{array}{*{20}{c}} . \\ {{\hat{\mathrm \Phi }}} \\ {} \end{array}} \hfill & = \hfill & { - \frac{{\hat Q}}{{C_0}} - \left( {\gamma _{\mathrm{t}} + \gamma _{\mathrm{r}}} \right){\hat{\mathrm \Phi }} - g_1\omega _{\mathrm{s}}L_0\hat Q\left( {\hat b + \hat b^\dagger } \right)} \hfill \\ {} \hfill & {} \hfill & { - g_2\omega _{\mathrm{s}}L_0\hat Q\left( {\hat n_{\mathrm{b}} + \frac{{\hat b\hat b + \hat b^\dagger \hat b^\dagger }}{2}} \right) + 2\left( {\hat V_{{\mathrm{in}}} + \hat V_{R_{0}}} \right)}, \hfill \end{array}$$

4 $$\begin{array}{*{20}{c}} . \\ {\hat b} \\ {} \end{array} = - {i}\omega _{\mathrm{m}}\hat b - g_1\frac{{{i}\omega _{\mathrm{s}}L_0\hat Q^2}}{{2\hbar }} - g_2\frac{{{i}\omega _{\mathrm{s}}L_0\hat Q^2}}{{2\hbar }}\left( {\hat b + \hat b^\dagger } \right) - \frac{{\gamma _{\mathrm{b}}}}{2}\hat b + {i}\frac{{x_0}}{\hbar }\hat F_{\mathrm{b}},$$

where *γ*_r_ = *R*_0_/*L*_0_, *γ*_b_ is the intrinsic mechanical damping rate with associated noise $\hat F_{\mathrm{b}}$ and *x*_0_ = $\sqrt {\hbar {\mathrm{/}}\left( {2m\omega _{\mathrm{m}}} \right)}$ is the amplitude of the zero-point motion for a membrane of mass *m*. From now on, we consider optimally loaded setups with *γ*_r_ = *γ*_t_. Equations (2)–(4) fully characterize the dynamics of the system, and represent the starting point for our detailed analysis.

The feedback of the membrane’s motion on the electrical circuit is described by Eq. (3). Driving the system at the electrical resonance frequency *ω*_s_, the terms proportional to $g_1 ( {\hat b + \hat b^\dagger } )$ and $g_2 ( {\hat b\hat b + \hat b^\dagger \hat b^\dagger } )$ give rise to sidebands at frequencies *ω*_s_ ± *ω*_m_ and *ω*_s_ ± 2*ω*_m_, respectively, whereas $g_2\hat n_{\mathrm{b}}$ induces a phonon-dependent frequency shift of the microwave cavity. Since homodyne detection is only sensitive to signals at the measured frequency, the sidebands are removed in the outcome *V*_M_, which is defined as the phase quadrature of $\hat V_{{\mathrm{out}}} = \hat V_{{\mathrm{in}}} - \gamma _{\mathrm{t}}{\hat{\mathrm \Phi }}$. This allows us to neglect oscillating terms in the calculation of *V*_M_ (the linear term also leads to mechanically induced damping, but this is typically negligible compared to *γ*_t_). The only contribution to *V*_M_ is therefore the phonon-dependent frequency shift, which allows us to resolve the mechanical state. On the contrary, the electrically induced mechanical heating only involves the sidebands *ω*_s_ ± *ω*_m_ and *ω*_s_ ± 2*ω*_m_, being unaffected by the term $g_2\hat n_{\mathrm{b}}$ in the Hamiltonian. For the *RLC* circuit in Fig. 1b, the heating is dominated by the linear term, since $g_1 \gg g_2$, and we shall neglect *g*_2_ for the calculation of Δ*n*_b_.

Below, we quantify the heating of the membrane and the phonon-dependent *LC* frequency shift. We first assume that the mechanical state does not jump during the measurement. Then, the equations of motion of the two subsystems decouple and we find *D*^2^ = $g_2^2\left| \alpha \right|^2{\mathrm{/}}\left[ {4(1 + 2\bar n_{\mathrm{e}})\gamma _{\mathrm{t}}^2} \right]$, where the number of photons $\left| \alpha \right|^2$ sent into the circuit within the measurement time *T* sets the measurement strength. As discussed above, Δ*n*_b_ is the average phonon number at the end of the measurement Δ*n*_b_ = $\left\langle {\hat n_{\mathrm{b}}(T)} \right\rangle$, with the mechanics initially in its ground state. For *T* much shorter than the mechanical lifetime $\gamma _{\mathrm{b}}^{ - 1}$, Δ*n*_b_ can be linearized to find the rate at which the membrane heats up. For the *RLC* circuit in Fig. 1b, we find Δ*n*_b_ = $\left( {1 + 2\bar n_{\mathrm{e}}} \right)g_1^2\left| \alpha \right|^2{\mathrm{/}}\left( {2\omega _{\mathrm{m}}^2} \right)$. The parameter *λ* given in Eq. (1) is then found as the ratio *λ* = *D*^2^/Δ*n*_b_. For details see Supplementary Note 1 available in  Supplementary Material online.

### Double-arm circuit

With the overall linear coupling vanishing, the parameter *λ* will be limited by fabrication imperfections and coupling to the antisymmetric mode. To model these phenomena, we consider the circuit in Fig. 1c, where the antisymmetric mode resides inside the small loop containing the two capacitors, and the symmetric one probes the system. We derive *g*_1_ and *g*_2_ from the expansion of each of the two capacitors: $1{\mathrm{/}}C\left( { \pm \hat x} \right)$ ≃ $C_0^{ - 1} \pm \tilde g_1 ( {\hat b + \hat b^\dagger } ) + \tilde g_2\hat n_{\mathrm{b}}$, so that in the absence of fabrication imperfections the total capacitor *C*_tot_ = $C\left( {\hat x} \right) + C\left( { - \hat x} \right)$ is not linearly coupled to the symmetric mode. The coefficients *g*_1_ and *g*_2_ are related to their tilde counterparts in the same way as before, and the parameters *D*^2^ and Δ*n*_b_ are evaluated in a similar fashion as we did for the *RLC* circuit. Since we quantify two sources of heating, it is convenient to write *λ* = $( {\lambda _{\mathrm{b}}^{ - 1} + \lambda _{\mathrm{p}}^{ - 1}} )^{ - 1}$, where *λ*_b_ takes into account heating from charge redistribution, and *λ*_p_ describes the influence of fabrication imperfections. With the details presented in Supplementary Note 2 (for details, see Supplementary Material available online) and Methods, we find

5 $$\lambda _{\mathrm{b}} = \frac{2}{{\left( {1 + 2\bar n_{\mathrm{e}}} \right)^2}}\left( {\frac{{g_2}}{{g_1}}} \right)^2\left( {\frac{{\omega _{\mathrm{s}}}}{{\gamma _{\mathrm{t}}}}} \right)^2\frac{{Z_{{\mathrm{out}}}}}{R},$$

6 $$\lambda _{\mathrm{p}} = \frac{2}{{\left( {1 + 2\bar n_{\mathrm{e}}} \right)^2}}\left( {\frac{{g_2}}{{g_1}}} \right)^2\left( {\frac{{g_1}}{{g_{\mathrm{r}}}}} \right)^2\left( {\frac{{\omega _{\mathrm{m}}}}{{\gamma _{\mathrm{t}}}}} \right)^2,$$

where *ω*_s_ = [*C*_0_(*L* + 2*L*_0_)]^−1/2^ is the frequency of the symmetric mode, *γ*_t_ = [2*Z*_out_]/[*L* + 2*L*_0_] is the decay to the transmission line and *g*_r_ = $2C_0x_0\omega _{\mathrm{s}}\partial _xC_{{\mathrm{tot}}}^{ - 1}(x)$ is the residual linear coupling induced by fabrication imperfection. We use the same notation introduced for the *RLC* circuit to allow a direct comparison. Equations (5) and (6) express the gain of our approach to QND detection. First, Eq. (6) quantifies the advantage of symmetry: *λ* dramatically improves compared to Eq. (1) by having a small residual linear coupling $g_{\mathrm{r}} \ll g_1$. Second, Eq. (5) is multiplied by the factor (*ω*_s_/*ω*_m_)^2^ with respect to Eq. (1). For microwave readout of a megahertz oscillator, this factor can be substantial. Furthermore, the mechnical oscillator is now only susceptible to the noise associated with charge redistribution on the capacitor, and not to the resistance in the inductor. This gives an additional improvement if *R* < *Z*_out_.

**Fig. 3:**
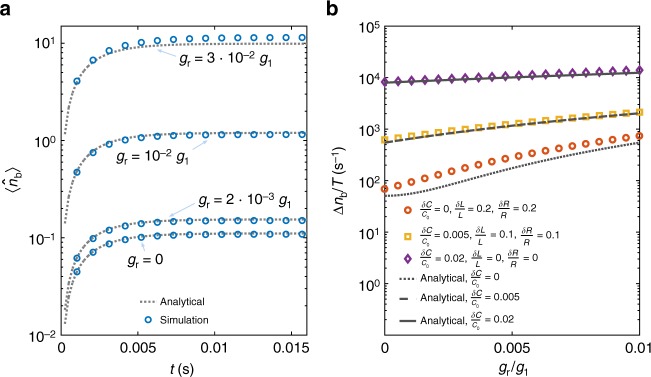
Heating simulations. **a** Average phonon number *n*_b_(*t*) as a function of time. We present a comparison between the analytical curves (grey, dotted lines) and the full simulations of the system (blue dots). From the bottom to the top we set *g*_r_/*g*_1_ to be 0, 2 × 10^−3^, 10^−2^ and 3 × 10^−2^. We use *δR* = *δL* = *δC* = 0. **b** Heating rate Δ*n*_b_/*T* as a function of the normalized residual linear coupling *g*_r_/*g*_1_. Here we analyse the system in the presence of asymmetries in the parasitic elements of the circuit. The three dark grey lines are the analytical predictions for *δC*/*C*_0_ being equal to 0 (dotted), 0.005 (dashed) and 0.02 (full). The circles, squares and diamonds are the simulated results for the values *δR*/*R*, *δL*/*L* and *δC*/*C* reported in the legend. We assume *L*/*L*_0_ = 10^−2^, *R*/*Z*_out_ = 10^−1^, *ω*_s_ = (2*π*)7 GHz, *ω*_m_ = (2*π*)80 MHz, *γ*_r_ ≃ *γ*_t_ = (2*π*)0.15 MHz, *γ*_b_ = (2*π*)80 Hz, *g*_1_ = (2*π*)7 kHz, $\bar n_{\mathrm{e}} = \bar n_{\mathrm{m}} = 0$, and an incident photon flux $\left| {\tilde \alpha } \right|^2$ = 1.15 × 10^15^ s^−1^

To describe a realistic situation, we numerically simulate the case in which the parasitic resistances *R*, inductances *L* and the two bare capacitances *C*_0_ differ from each other. In Fig. 3, we test the system with these asymmetries and the physical parameters given below. In the left plot, the role of a residual linear coupling *g*_r_ is investigated. In the right one, we consider unbalanced resistances *R* ± *δR*, inductances *L* ± *δL* and capacitances *C*_0_ ± *δC*. The results show that our analytical predictions accurately describe a system with non-zero *g*_r_ and *δC*. Furthermore, the numerical points confirm that *δR* and *δL* enter as higher order perturbations. In fact, we generally find that Eqs. (5) and (6) are accurate for relatively large perturbations (up to 25%).

Inspired by recent experiments^15,35–38^, we estimate the value of *λ*, which can be reached in state-of-the-art setups. We consider a rectangular monolayer graphene membrane of length 1 μm and width 0.3 μm, with a mechanical frequency of *ω*_m_ = (2*π*)80 MHz and a quality factor *Q* = 10^6^. It is suspended *d*_0_ = 10 nm above a conducting plate, forming the capacitor (see sketch in Fig. 1a). Assuming that the membrane is clamped to the substrate along its boundaries, we identify the ratio of the coupling coefficients for each capacitor $C( \pm \hat x)$ in Fig. 1c to be *g*_2_/*g*_1_ = $\pi ^2x_0{\mathrm{/}}\left( {8d_0} \right)$^39^. Considering that for these geometries stray capacitances *C*_s_ are typically preponderant with respect to *C*_0_, we take *g*_1_ ≃ (2*π*)7 kHz and *g*_2_ ≃ (2*π*)1 Hz, corresponding to *C*_s_ ≃ 100*C*_0_. For comparison, a value of *C*_s_ = 50 fF is obtained in ref. ^35^, for a graphene membrane about two and a half times the size considered here. This stray capacitance would be 376 times *C*_0_ ≃ 13 fF. Assuming a reduction of *C*_s_ due to the smaller dimensions, we take *C*_s_ = 100*C*_0_.

**Fig. 4:**
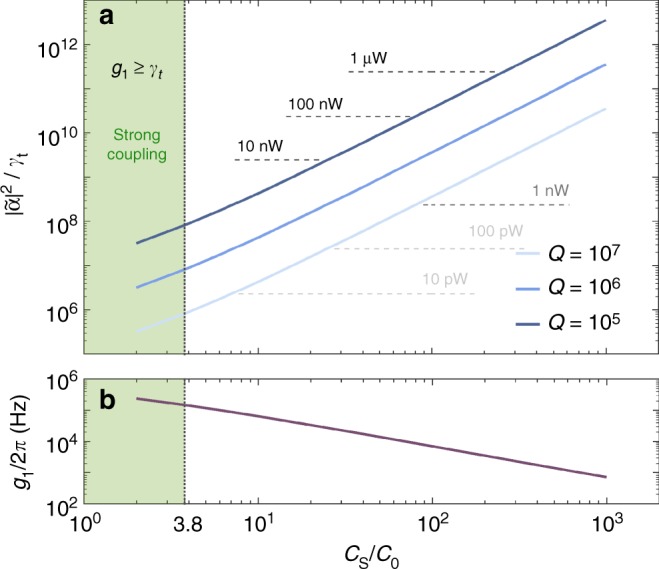
Operating conditions in the presence of stray capacitances. **a** Average intracavity photons $\left| {\tilde \alpha } \right|^2{\mathrm{/}}\gamma _{\mathrm{t}}$ required for the QND measurement, as a function of the relative value of the stray capacitance *C*_s_/*C*_0_. The three lines correspond to different values of the mechanical quality factor, as indicated in the legend. We assume Δ*n*_b_ = 0.3 and equal contributions from the mechanical and electrically induced reservoirs $\bar n_{\mathrm{m}} = \bar N_{{\mathrm{eff}}}{\mathrm{/}}2 = 3$. As a reference, the grey dashed lines indicate the associated powers of the probe. **b** Linear coupling *g*_1_ as a function of *C*_s_/*C*_0_. For both figures, the shadowed region indicates the strong coupling *g*_1_ ≥ *γ*_t_, where QND detection is feasible with other approaches^26, 31, 32^

With an electrical reservoir at zero temperature $\bar n_{\mathrm{e}}$ ≃ 0 (valid for milliKelvin experiments), an electrical frequency *ω*_s_ = (2*π*)7 GHz and decay rate *γ*_t_ = (2*π*)150 kHz, we get *λ*_b_ = 105 × *Z*_out_/*R* and *λ*_p_ = 0.014 × (*g*_1_/*g*_r_)^2^. Since the graphene coupling can be tuned via electric fields^40–42^, we assume *g*_1_/*g*_r_ ~ 100, which fixes *λ* between 60 (*R* = *Z*_out_) and 122 (*R* = *Z*_out_/10), mostly restricted by *λ*_p_. This limit is well above the threshold for having a good visibility of the phonon number states (see below), and can be further improved by either increasing the sideband resolution *ω*_m_/*γ*_t_, the electrical frequency *ω*_s_ or by reducing the size of the membrane. In Fig. 4b, we show the linear coupling *g*_1_ as a function of the stray capacitance. For small values of *C*_s_, we reach the strong-coupling regime, where *g*_1_ ≥ *γ*_t_. In the realistic scenario described above, where $C_{\mathrm{s}} \gg C_0$, our scheme still allows for phonon QND measurement even for $g_1,g_2 \ll \gamma _t$. This is in contrast to the typical optomechanical approach, where the quadratic interaction results from a hybridization of two optical modes, and strong coupling *g*_1_ > *γ*_t_ is required^28^. Regardless of how much *C*_*s*_ reduces the coupling constants, it is in principle always possible to compensate by using stronger power. For details see the Supplementary Note 4 available in Supplementary Material online).

### Measurement

We now evaluate how well a given value of *λ* allows for the QND detection of the phonon number. To this end, we consider a situation where the system is continuosly probed and measured. The output is then turned into discrete results by averaging over a suitable time *T*, and a histogram is constructed from the measured values *V*_M_. We assume that the heating of the continuous QND probing is in equilibrium with the mechanical damping and the associated reservoir. In this case, one also needs to consider the thermal bath of the membrane. In addition to Δ*n*_b_ determined above, the total heating out of the ground state is thus Δ*n*_b_ + *γ*_b_$\bar n_{\mathrm{m}}$*T*. This additional term leads to a redefinition of the parameter *λ* to

7 $$\lambda \prime = \lambda \frac{{{\mathrm{\Delta }}n_{\mathrm{b}}}}{{{\mathrm{\Delta }}n_{\mathrm{b}} + \gamma _{\mathrm{b}}\bar n_{\mathrm{m}}T}},$$

and the equilibrium average mechanical occupation, resulting from both the mechanical reservoir and the QND probe, becomes

8 $$\bar N_{{\mathrm{eff}}} \simeq \bar n_{\mathrm{m}}\frac{\lambda }{{\lambda - \lambda \prime }}.$$

**Fig. 5:**
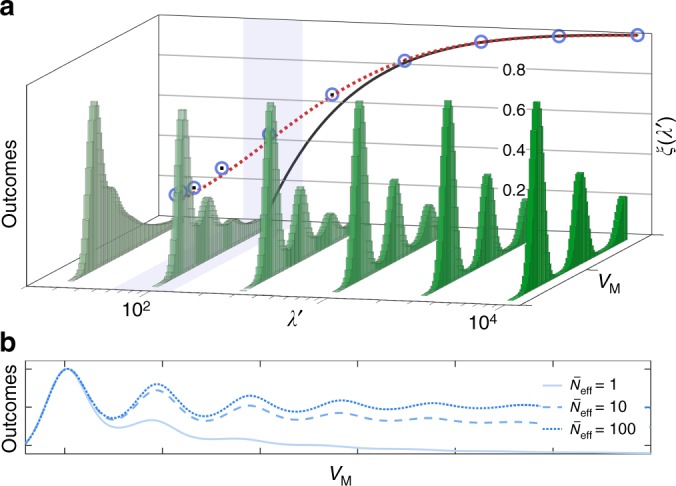
Visibility of the phonon QND measurement. **a** 3D plot: histograms of outcomes for different *λ*′ (from left to right, *λ*′ = 32, 10^2^, 3 × 10^2^, 10^3^, 3 × 10^3^ and 10^4^). The optimal values of Δ*n*_b_ are (from left to right) 0.43, 0.27, 0.12, 0.05, 2 × 10^−3^ and 8 × 10^−4^, and have been determined by a numerical optimization. The shadowed region corresponds to the estimated visibility for state-of-the-art technology, *λ* ≃ 60–130. 2D plot (back): maximum visibility *ξ* for different values of the parameter *λ*′. The blue circles have been evaluated numerically from the histograms in the 3D plot (and others). The error bars of the Monte Carlo simulation (black lines inside) have been determined assuming Poissonian statistics in each bin, and are negligible on this scale. The red dotted curve comes from our model for the visibility, and the black solid curve is the simplified expression presented in Eq. (10). We consider $\bar N_{{\mathrm{eff}}} = 1$. See Methods for more details. **b** Expected outcomes for *λ*′ = 75 and $\bar N_{{\mathrm{eff}}}$ being 1 (full), 10 (dashed) and 100 (dotted line). The parameter Δ*n*_b_ has been optimized to achieve maximum visibility for each value of $\bar N_{{\mathrm{eff}}}$

The phonon QND measurement is then characterized by *λ*′, which is desirable to have as close as possible to its maximum *λ*. This can be achieved by choosing a sufficiently strong probing power and a short measurement time *T*, such that the mechanical heating can be neglected. This leads to a large $\bar N_{{\mathrm{eff}}}$, which does not significantly change the contrast of the QND measurement (see Eq. (10) and Fig. 5b), but increases the time for acquiring significant statistics (the mechanical system spends less time in each Fock state).

Given *λ*′, we now want to optimize all remaining parameters of the system, to be able to discern the ground and first excited states with the largest contrast. We simulate the mechanical system with the quantum-jump method, and pick Gaussian distributed random values for the electrical vacuum and thermal noise. From this, we make the histogram of the resulting output voltages *V*_M_ presented in Fig. 5a, where the induced heating Δ*n*_b_ is optimized numerically. For the optimization we consider the visibility

9 $$\xi = \frac{{\frac{1}{2}\left( {I_0 + I_1} \right) - I_{\mathrm{R}}}}{{\frac{1}{2}\left( {I_0 + I_1} \right) + I_{\mathrm{R}}}},$$

where *I*_0_ and *I*_1_ are the heights of the peaks corresponding to *n*_b_ = 0 and *n*_b_ = 1 phonons, while *I*_R_ is the lowest height in between *I*_0_ and *I*_1_ (see Fig. 2).

Additionally, we make an analytical model where we allow for one jump during each measurement period. We can extract the asymptotic behaviour of the visibility

10 $$\xi \left( {\lambda \prime ,\bar N_{{\mathrm{eff}}}} \right) = 1 - 8\frac{{3 + 5\bar N_{{\mathrm{eff}}}}}{{1 + 2\bar N_{{\mathrm{eff}}}}}\frac{{\sqrt {\pi {\kern 1pt} {\mathrm{log}}{\kern 1pt} \lambda \prime } }}{{\lambda \prime }},$$

reflecting the compromise between the contributions to *I*_R_ from the noise ∝ exp(−*D*^2^/8) and from the jumps during the measurements ∝ Δ*n*_b_.

The results of simulations and model are shown in Fig. 5a. The blue points are the numerical optimization, which are in good agreement with the analytical result (red, dotted line). Notice that for small values of *λ*′, the optimal Δ*n*_b_ is sufficiently high to allow multiple jumps during the measurement time *T*, leading to minor discrepancies. The black, solid line is Eq. (10), and the shadowed region corresponds to the predicted values of *λ* for the parameters introduced above. Qualitatively, clear signatures of the mechanical energy quantization are present for *λ*′ ≳ 40, where the visibility exceeds 20%.

For the experimental parameters considered above, the maximum attainable value of *λ*′ is *λ* = 122 (for *R* = *Z*_out_/10), and is achieved with a strong probe such that $\bar N_{{\mathrm{eff}}} \gg \bar n_{\mathrm{m}}$. The incident power and the measurement time *T* provide a handle to optimize the performance for given experimental conditions. Qualitatively, a short value of *T* minimizes the effects of the mechanical heating, and makes *λ*′ ≃ *λ*. On the other hand, the required power to reach such a regime can be troublesome^43^, and we may need to integrate for too long time to have sufficient statistics (since $\bar N_{{\mathrm{eff}}} \gg 1$). This last problem can be solved by adding an electrical cooling, red-detuned by $\omega _{\mathrm{m}} \gg \gamma _{\mathrm{t}}$ from the QND probe. This cooling would not affect the parameter *λ*′, since it does not heat up the system, but only reduces $\bar N_{{\mathrm{eff}}}$. The visibility *ξ* thus remains almost unaltered (see Eq. (10) and Fig. 5b), but the probability to find the membrane in low excited states is increased, reducing the experimental time.

As an example, assume that the heating from the electrical feedback and the mechanical bath are equal, such that *λ*′ = *λ*/2 = 61. Considering a cryogenic temperature of 14 mK^37^, the average mechanical occupation is $\bar n_{\mathrm{m}}$ ≃ 3, implying $\bar N_{{\mathrm{eff}}} = 6$. The optimal Δ*n*_b_ is then 0.3, and can be obtained with a driving power of 16 nW and a measurement time of 0.1 ms for a mechanical quality factor *Q* = 10^6^ and a stray capacitance *C*_s_ = 100*C*_0_. For other values of *Q* and *C*_s_, the driving power can be varied to fulfil the constraint $\bar N_{{\mathrm{eff}}} = 2\bar n_{\mathrm{m}}$, as shown in Fig. 4a. The incident field is rather intense, which may cause additional heating to the system. In the set up of ref. ^43^, such additional heating has been observed above an intracavity photon number of 10^8^. For comparison, in Fig. 4a we show the intracavity photon number $\left| {\tilde \alpha } \right|^2{\mathrm{/}}\gamma _{\mathrm{t}}$ for our system, where $\left| {\tilde \alpha } \right|^2 = \left| \alpha \right|^2{\mathrm{/}}T$ is the photon flux. Depending on the parameters, we see that $\left| {\tilde \alpha } \right|^2{\mathrm{/}}\gamma _{\mathrm{t}}$ will be similar or higher than 10^8^ for *C*_s_ ≳ 100*C*_0_. These devices cannot, however, be compared directly. Nevertheless, since ref. ^43^ indicates that the source of this heating is electrical, we believe that it would be strongly suppressed for the QND measurement considered here. Since the linear coupling is almost cancelled by symmetry, the resulting heating rate is likely reduced by a factor (*g*_*r*_/*g*_1_)^2^ ≃ 10^−4^. In absence of this suppression, conducting our experiment in a pulsed regime may substantially reduce other heating mechanisms.

## Discussion

We have revisited the challenge of performing a phonon QND measurement. Employing symmetry to inhibit the linear coupling, the detrimental heating is suppressed while retaining the desired quadratic coupling. Contrary to the generally studied optomechanical case^28^, the residual coupling to the antisymmetric mode is strongly suppressed by its higher frequency and reduced resistance. A particularly attractive feature of the current approach is that it is only sensitive to the ratio *g*_2_/*g*_1_, and not to their absolute values. Stray capacitances, which reduce the electromechanical couplings, can thus be compensated using stronger input fields.

These attractive features put QND detection within reach of presently available technology. A successful realization of a QND detection will not only represent a demonstration of genuine non-classical behaviour of mechanical systems, but also extend the interactions available in electro/optomechanics to non-Gaussian operations^44^. This will considerably expand the realm of effects that can be studied with these systems, and facilitate their application for quantum information processing^23^.

As an outlook, it is desirable to extend this work to the optomechanical case, where mechanical systems with a similar quadratic coupling have recently been studied^33,45,46^, but conditions to have a successful phonon QND measurement have not been yet determined. The electromechanical systems considered here can be described with Kirchoff’s laws, which give rigorous results within a well-defined model. The physical mechanisms behind the heating are identified to be the Johnson–Nyquist noises associated to the resistors, and fabrication imperfections. For comparison, the exact description of dissipation in a multimode optomechanical system may be more involved. Nevertheless, the results presented here could be useful for guiding the intuition towards QND detection in the optical regime. As a further extension, it would be interesting to investigate the effect of squeezing. By reducing the vacuum noise, squeezing can lead to a direct improvement in *λ*, thus reducing the physical requirements for the QND detection.

## Methods

### The double-arm circuit

The Hamiltonian for the system in Fig. 1c is given by

11 $$\begin{array}{*{20}{l}} {\hat {\cal H}} \hfill & = \hfill & {\hbar \omega _{\mathrm{m}}\hat b^\dagger \hat b + \frac{{{\hat{\mathrm \Phi }}_{\mathrm{a}}^2}}{{4L}} + \frac{{\hat Q_{\mathrm{a}}^2}}{{C_0}} + \frac{{{\hat{\mathrm \Phi }}_{\mathrm{s}}^2}}{{L + 2L_0}} + \frac{{\hat Q_{\mathrm{s}}^2}}{{4C_0}}} \hfill \\ {} \hfill & {} \hfill & { + \frac{{g_1}}{{C_0\omega _{\mathrm{s}}}}\hat Q_{\mathrm{a}}\hat Q_{\mathrm{s}} ( {\hat b + \hat b^\dagger } ) + \frac{{g_2}}{{C_0\omega _{\mathrm{s}}}}\hat Q_{\mathrm{a}}^2\hat b^\dagger \hat b + \frac{{g_2}}{{4C_0\omega _{\mathrm{s}}}}\hat Q_{\mathrm{s}}^2\hat b^\dagger \hat b,} \hfill \end{array}$$

where subscripts “a” and “s” indicate the asymmetric and the symmetric electrical fields, respectively. From Eq. (11) and using Kirchoff’s laws, it is possible to determine the equations of motions, including noises and decays. The normalized distance *D*^2^ = *d*^2^/*σ*^2^ is obtained assuming the phonon number to be constant within *T*—that is: setting *g*_1_ = 0—so that the asymmetric and symmetric fields decouple. Looking at the phase quadrature of the reflected signal $\hat V_{{\mathrm{out}}} = \hat V_{{\mathrm{in}}} - \gamma _{\mathrm{t}}{\hat{\mathrm \Phi }}_{\mathrm{s}}$, we determine *d*. The noise *σ* is the sum of vacuum noise from the input coherent field, and the Johnson–Nyquist noises of the resistors.

As discussed above, the heating ${\mathrm{\Delta }}n_{\mathrm{b}} = \left\langle {\hat n_{\mathrm{b}}(T)} \right\rangle$ has two contributions: asymmetries leading to a non-vanishing linear coupling *g*_r_, and the charge redistribution. The first is found by assuming $R \ll R_0$ and $L \ll L_0$, such that the circuit in Fig. 1c is equivalent to the one in Fig. 1b, for which we already know Δ*n*_b_. The contribution from charge redistribution is determined from the Hamiltonian in Eq. (11) neglecting the quadratic interaction, which does not alter the phonon number. The strongly driven symmetric electrical field is then substituted with its steady state, obtained assuming a constant photon flux. The time evolution of $\left\langle {\hat n_{\mathrm{b}}} \right\rangle$ is finally found by looking at the equations of motion for the asymmetric field and the mechanical creation/annihilation operators. With the amplitude of the symmetric mode replaced by its steady state, these equations are now linear in the annihilation (creation) operators $\hat b$ $( {\hat b^\dagger })$ and can be solved by standard optomechanics techniques.

### Asymmetric circuit

To obtain Fig. 3, we analyse the system in the presence of asymmetries. First, we derive the generalization of the Hamiltonian in Eq. (11) with unequal rest capacitors, resistors, inductors and linear couplings. Differently from above, we linearize the symmetric/asymmetric electrical fields around their mean values ($\hat Q_{\mathrm{a/s}} \to \langle {\hat Q_{\mathrm{a/s}}} \rangle + \hat \delta Q_{\mathrm{a/s}}$ and $\hat \phi _{\mathrm{a/s}} \to \langle {\hat \phi _{\mathrm{a/s}}} \rangle + \hat \delta \phi _{\mathrm{a/s}}$), and the mechanical creation/annihilation operators $( {\hat b^{(\dagger )} \to \langle {\hat b^{(\dagger )}} \rangle + \hat \delta b^{(\dagger )}} )$. Here, besides the usual oscillatory behaviour of the mechanical operators $\langle {\hat b^{(\dagger )}} \rangle$, the amplitude is generally time dependent^47^. This can be understood by looking at Eq. (11); since both the electrical fields have now non-zero average, the three body interaction $\propto \hat Q_{\mathrm{a}}\hat Q_{\mathrm{s}} ( {\hat b + \hat b^\dagger } )$ is equivalent to a force directly driving the mechanical system. Once solutions for the averages are found, it is possible to determine the variations, and finally the time evolution of the phonon number.

### Optimization of the visibility

To obtain Fig. 5 we rely on both an analytical and a numerical optimization of the visibility *ξ*. To determine the red, dotted curve, we assume that the initial mechanical state is thermal, such that the occupations of the Fock states can be found. Given Δ*n*_b_, the probability to jump once either up or down during the measurement time *T* is a Poissonian process. The probability distribution function for the outcomes *V*_M_ can then be obtained and maximized, by varying Δ*n*_b_. The histograms and the blue points are derived with Monte Carlo simulations, where the time evolution of single mechanical trajectories are replicated with the stochastic wave-function method^48^. Importantly, every measurement interval of duration *T* has been discretized, to allow for multiple jumps. The parameter Δ*n*_b_ is then varied to find the best visibility *ξ*.

## Electronic supplementary material

Supplementary Information

Peer Review File

## Data Availability

All material related to this work can be found at https://sid.erda.dk/share_redirect/eUaGoI8JbN.
