# Supplementary material for: Quantum nondemolition measurement of mechanical motion quanta
Source: Nat Commun. 2018 Sep 6;9:3621. doi: 10.1038/s41467-018-06070-y (PMC6127154; doi:10.1038/s41467-018-06070-y)
Supplement: Supplementary file 1 — Supplementary Information [file 41467_2018_6070_MOESM1_ESM.pdf]

Supplementary Information: Quantum nondemolition measurement of mechanical motion quanta

Luca Dellantonio,[1,2] Oleksandr Kyriienko,[1,3] Florian Marquardt,[4,5] and Anders S. Sørensen[1,2]

[1] *The Niels Bohr Institute, University of Copenhagen, Blegdamsvej 17, DK-2100 Copenhagen Ø, Denmark*

[2] *Center for Hybrid Quantum Networks (Hy-Q), Niels Bohr Institute, University of Copenhagen, Blegdamsvej 17, DK-2100 Copenhagen Ø, Denmark*

[3] *NORDITA, KTH Royal Institute of Technology and Stockholm University, Roslagstullsbacken 23, SE-106 91 Stockholm, Sweden*

[4] *Institute for Theoretical Physics, University Erlangen-Nürnberg, Staudstraße 7, 91058 Erlangen, Germany*

[5] *Max Planck Institute for the Science of Light, Günther-Scharowsky-Straße 1, 91058 Erlangen, Germany*

(Dated: July 26, 2018)

SUPPLEMENTARY NOTE 1

Single arm circuit

In the following we consider an RLC circuit where the capacitor contains an oscillating element. Following the standard procedure for quantizing an electrical circuit [1], we can write the Hamiltonian of the setup presented in supplementary Fig. 1 as

$$\hat{\mathcal{H}} = \hbar\omega_{\rm m}\hat{b}^\dagger\hat{b} + \frac{\hat{\Phi}^2}{2L_0} + \frac{\hat{Q}^2}{2C_0} + \frac{g_1\omega_{\rm s}L_0}{2}\hat{Q}^2(\hat{b}+\hat{b}^\dagger) + \frac{g_2\omega_{\rm s}L_0}{2}\hat{Q}^2\left(\hat{b}^\dagger\hat{b} + \frac{\hat{b}\hat{b}+\hat{b}^\dagger\hat{b}^\dagger}{2}\right) - 2\hat{Q}(\hat{V}_{\rm in}+\hat{V}_{\rm R0}) - x_0\hat{F}_{\rm b}(\hat{b}+\hat{b}^\dagger), \quad (1)$$

where the conjugate position $\hat{Q}$ and momentum $\hat{\Phi} = L_0\hat{I}$ are the electrical charge and flux, respectively. $x_0 = \sqrt{\hbar/(2m\omega_{\rm m})}$ is the zero–point motion amplitude for a membrane of mass m, and $\hat{b}$ ($\hat{b}^\dagger$) denotes the mechanical annihilation (creation) operator. $\omega_{\rm m}$ and $\omega_{\rm s} = (C_0L_0)^{-\frac{1}{2}}$ are the mechanical and electrical resonance frequencies. C_0, g_1 and g_2 are derived from the expansion of the inverse capacitance,

$$C^{-1}(\hat{b}+\hat{b}^\dagger) \simeq C_0^{-1} + g_1L_0\omega_{\rm s}(\hat{b}+\hat{b}^\dagger) + g_2L_0\omega_{\rm s}\left(\hat{b}+\hat{b}^\dagger\right)^2, \quad (2)$$

where the coupling required for the QND interaction comes from the rotating wave approximation: $(\hat{b}+\hat{b}^\dagger)^2 \simeq 2\hat{n}_{\rm b}+1$. Finally, $\hat{V}_{\rm in}$ is the input field, $\hat{V}_{\rm R0}$ is the Johnson–Nyquist noise associated with the resistor R_0, and $\hat{F}_{\rm b}$ is the random force related to the mechanical reservoir that slowly thermalizes the membrane. From Supplementary Eq. (1) we can

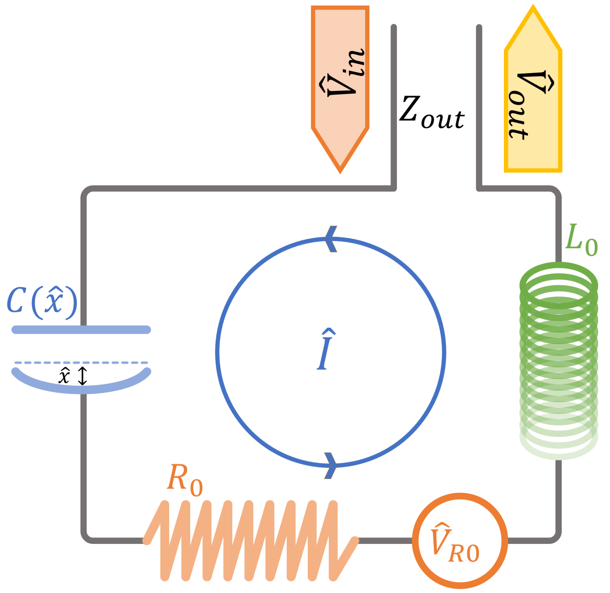

Supplementary Fig. 1: **Single arm circuit.** Circuit diagram for an RLC circuit coupled capacitively to a mechanical oscillator, with the position operator denoted as $\hat{x}$. R_0 and L_0 are the inductance and resistance of the electrical circuit, while $C(\hat{x})$ is the position–dependent capacitance. The setup is driven by the microwave (MW) field $\hat{V}_{\rm in}$ through the semi–infinite transmission line of impedance $Z_{\rm out}$. $\hat{V}_{\rm out}$ is the reflected signal, $\hat{V}_{\rm R0}$ the Johnson–Nyquist noise associated with R_0, and $\hat{I}$ is the current flowing in the circuit.

derive the Heisenberg equations of motion for the electromechanical system operators $\hat{Q}$, $\hat{\Phi}$ and $\hat{b}$. Adding decays and noises to the equations of motion we have

$$\dot{\hat{Q}} = \frac{\hat{\Phi}}{L_0}, \quad (3)$$

$$\dot{\hat{\Phi}} = -\frac{\hat{Q}}{C_0} - g_1\omega_{\rm s}L_0\hat{Q}\left(\hat{b}+\hat{b}^\dagger\right) - g_2\omega_{\rm s}L_0\hat{Q}\left(\hat{n}_{\rm b} + \frac{\hat{b}\hat{b}+\hat{b}^\dagger\hat{b}^\dagger}{2}\right) - (\gamma_{\rm t}+\gamma_{\rm r})\hat{\Phi} + 2\left(\hat{V}_{\rm in}+\hat{V}_{\rm R0}\right), \quad (4)$$

$$\dot{\hat{b}} = -i\omega_{\rm m}\hat{b} - g_1\frac{i\omega_{\rm s}L_0\hat{Q}^2}{2\hbar} - g_2\frac{i\omega_{\rm s}L_0\hat{Q}^2}{2\hbar}\left(\hat{b}+\hat{b}^\dagger\right) - \frac{\gamma_{\rm b}}{2}\hat{b} + i\frac{x_0}{\hbar}\hat{F}_{\rm b}. \tag{5}$$

Importantly, these expressions are just the familiar Kirchoff's laws, which provide the form of the electrical decay rates $\gamma_{\rm r} = R_0/L_0$ and $\gamma_{\rm t} = Z_{\rm out}/L_0$. The mechanical decay rate $\gamma_{\rm b}$ is an intrinsic property of the membrane, and we have assumed that the mechanical reservoir can be treated using the Markov approximation. Finally, the reflected signal is determined by the input/output relations

$$\hat{V}_{\rm out} = \hat{V}_{\rm in} - \gamma_{\rm t}\hat{\Phi}, \tag{6}$$

that read the same in the time and frequency domains.

QND measurement of the phonon number

As explained in the main text, there are three mechanisms with which the mechanical system influences the electrical one. These are the two terms $g_1(\hat{b}+\hat{b}^\dagger)$ and $g_2(\hat{b}\hat{b}+\hat{b}^\dagger\hat{b}^\dagger)$, which generate sidebands at frequencies $\omega_{\rm s}\pm\omega_{\rm m}$ and $\omega_{\rm s}\pm 2\omega_{\rm m}$, and a phonon–dependent frequency shift proportional to $g_2\hat{n}_{\rm b}$ [see Supplementary Eq. (4)]. When we perform homodyne measurement at the resonant frequency of the electrical circuit $\omega_{\rm s}$, the sidebands contribution to the measurement outcome averages out, and we are left with the phonon–dependent frequency shift. The main detrimental effect of g_1 will be to cause heating. We consider this later in this Note, and in the following discussion of the readout we thus assume that the phonon number is conserved, $\hat{n}_{\rm b}(t)\rightarrow\hat{n}_{\rm b} = \text{constant}$. Under these assumptions, the electrical readout is independent of the mechanical dynamics, and can be described by

$$\dot{\hat{Q}} = \frac{\hat{\Phi}}{L_0}, \tag{7}$$

$$\dot{\hat{\Phi}} = -\frac{\hat{Q}}{C_0} - g_2\omega_{\rm s}L_0\hat{Q}\hat{n}_{\rm b} - (\gamma_{\rm t}+\gamma_{\rm r})\hat{\Phi} + 2\left(\hat{V}_{\rm in}+\hat{V}_{\rm R0}\right). \tag{8}$$

As it is instructive to look at these equations in the frequency domain, we introduce the Fourier series of any operator $\hat{O}(t)$ by

$$\hat{O}(t) = \sum_{k=-\infty}^{\infty}\hat{O}[\Omega_{\rm k}]\frac{e^{-i\Omega_{\rm k}t}}{\sqrt{\tau}}, \tag{9}$$

with $\Omega_{\rm k} = 2\pi k/\tau$ ($k\in\mathbb{Z}$) being the allowed frequencies, and τ is the time period used to define the Fourier series. For now we will let τ be equal to the measurement time T, but later we shall consider a larger value to describe heating on longer time scales. The Fourier coefficients $\hat{O}[\Omega_{\rm k}]$ are then defined by

$$\hat{O}[\Omega_{\rm k}] = \int_0^\tau \hat{O}(t)\frac{e^{i\Omega_{\rm k}t}}{\sqrt{\tau}}dt. \tag{10}$$

It is possible to rewrite equations (3), (4) and (5) in the frequency domain:

$$-i\Omega_{\rm k}\hat{\Phi}[\Omega_{\rm k}] = -\frac{\hat{Q}[\Omega_{\rm k}]}{C_0} - g_2\omega_{\rm s}L_0\hat{Q}[\Omega_{\rm k}]\hat{n}_{\rm b} - (\gamma_{\rm r}+\gamma_{\rm t})\hat{\Phi}[\Omega_{\rm k}] + 2(\hat{V}_{\rm in}[\Omega_{\rm k}]+\hat{V}_{\rm R0}[\Omega_{\rm k}]), \tag{11}$$

$$-i\Omega_{\rm k}\hat{Q}[\Omega_{\rm k}] = \frac{\hat{\Phi}[\Omega_{\rm k}]}{L_0}. \tag{12}$$

From these two relations we derive $\hat{\Phi}[\Omega_{\rm k}]$, which can then be used in Supplementary Eq. (6) for determining $\hat{V}_{\rm out}[\Omega_{\rm k}]$:

$$\hat{V}_{\rm out}[\Omega_{\rm k}] = \left[1-\zeta(\Omega_{\rm k},\hat{n}_{\rm b})\right]\hat{V}_{\rm in}[\Omega_{\rm k}] - \zeta(\Omega_{\rm k},\hat{n}_{\rm b})\hat{V}_{\rm R0}[\Omega_{\rm k}], \tag{13}$$

$$\zeta(\Omega_{\rm k},\hat{n}_{\rm b}) = \frac{2\gamma_{\rm t}\Omega_{\rm k}}{-i(\Omega_{\rm k}^2-\omega_{\rm s}^2-g_2\omega_{\rm s}\hat{n}_{\rm b}) + \Omega_{\rm k}(\gamma_{\rm r}+\gamma_{\rm t})}. \tag{14}$$

These expressions describe the principle of the QND measurement: the quadratic interaction shifts the resonance frequency of the circuit by an amount proportional to $g_2 n_{\rm b}$ (denominator of Supplementary Eq. (14)). By sending a signal resonant with the electrical circuit, it is possible to detect this frequency change as a phase shift of the reflected signal.

From the quantized form of $\hat{V}_{\rm in}[\Omega_{\rm k}]$ and $\hat{V}_{\rm R0}[\Omega_{\rm k}]$ [2], we have the Fourier components

$$\hat{V}_{\rm in}[\Omega_{\rm k}] = \sqrt{\frac{\hbar\Omega_{\rm k} Z_{\rm out}}{2}}\hat{a}_{\rm in,k}, \tag{15}$$

$$\hat{V}_{\rm R0}[\Omega_{\rm k}] = \sqrt{\frac{\hbar\Omega_{\rm k} R_0}{2}}\hat{a}_{\rm R0,k}, \tag{16}$$

where $\hat{a}_{\rm in,k}$ and $\hat{a}_{\rm R0,k}$ are the annihilation operators for the input field and the electrical reservoir, and satisfy the standard commutation relations. The measurement outcome $\hat{V}_{\rm M}$ is obtained by homodyne detection, meaning that we have access to

$$\hat{V}_{\rm M}[\Omega_{\rm k}] = \frac{e^{i\frac{\theta}{2}}\hat{V}_{\rm out}[\Omega_{\rm k}] + e^{-i\frac{\theta}{2}}\hat{V}^{\dagger}_{\rm out}[\Omega_{\rm k}]}{2}, \tag{17}$$

where θ is an arbitrary phase that allows choosing the quadrature of the reflected signal to be measured.

Having now all the operator equations describing the system in the Heisenberg formalism, we need to define the input state $\hat{\rho}_{\rm in}$. Assuming that the semi–infinite transmission line of impedance $Z_{\rm out}$ and the resistor R_0 are connected to reservoirs at the same temperature $T_{\rm e}$, and that we drive the circuit with a coherent field at the resonant frequency $\omega_{\rm s}$, we have:

$$\hat{\rho}_{\rm in}(\tilde{\alpha}, \beta_{\rm R}) = \frac{1}{\mathbf{Z}}\hat{D}(\tilde{\alpha})e^{-\beta_{\rm R}\hat{\mathcal{H}}_{\rm R}}\hat{D}^{\dagger}(\tilde{\alpha}). \tag{18}$$

Here, $\mathbf{Z} = {\rm Tr}\left\{e^{-\beta_{\rm R}\hat{\mathcal{H}}_{\rm R}}\right\}$ is the partition function, $\hat{D}(\tilde{\alpha}) = \exp\left(\int_0^T \tilde{\alpha}(t)\hat{a}^{\dagger}_{in,s}(t) - \tilde{\alpha}^*(t)\hat{a}_{in,s}(t)dt\right)$ is the displacement operator, $|\tilde{\alpha}|^2$ is the photon flux, $\hat{\mathcal{H}}_{\rm R} = \sum_{\rm k}\left\{\hbar\Omega_{\rm k}\hat{a}^{\dagger}_{\rm in,k}\hat{a}_{\rm in,k} + \hbar\Omega_{\rm k}\hat{a}^{\dagger}_{\rm R0,k}\hat{a}_{\rm R0,k}\right\}$ is the reservoir Hamiltonian, and $\beta_{\rm R}^{-1} = k_{\rm b}T_{\rm e}$ with $k_{\rm b}$ being the Boltzmann constant. Using $\hat{\rho}_{\rm in}(\tilde{\alpha}, \beta_{\rm R})$ it is possible to calculate the average measured signal $V_{\rm M} = \langle\hat{V}_{\rm M}\rangle$ and the variance $(\Delta\hat{V}_{\rm M})^2 = \langle\hat{V}^2_{\rm M}\rangle - \langle\hat{V}_{\rm M}\rangle^2$ for an incident field at the resonance frequency $\omega_{\rm s}$:

$$V_{\rm M}(n_{\rm b}) = -\alpha\sqrt{\frac{\hbar\omega_{\rm s}Z_{\rm out}}{2}}{\rm Im}\{\zeta(\omega_{\rm s}, n_{\rm b})\}, \tag{19}$$

$$(\Delta\hat{V}_{\rm M})^2 = \frac{\hbar\omega_{\rm s}Z_{\rm out}}{2}\left[1 + 2\bar{n}_{\rm e}(\omega_{\rm s}, T_{\rm e})\right], \tag{20}$$

where $|\alpha|^2 = \int_0^T|\tilde{\alpha}(t)|^2dt$ is the number of photons sent into the circuit during the measurement. The imaginary part is denoted with ${\rm Im}\{\cdot\}$, and $\bar{n}_{\rm e}(\omega_{\rm s}, T_{\rm e}) = \left[\exp\left(\hbar\beta_{\rm R}\omega_{\rm s}\right) - 1\right]^{-1}$ is the average number of photons at frequency $\omega_{\rm s}$ in the electrical reservoir. Note that we have taken $\theta = \pi$ in Supplementary Eq. (17), and $\tilde{\alpha}(t) = \alpha/\sqrt{T}$ to be purely real. With this choice, we measure the phase quadrature, which optimizes the signal for small g_2 (see supplementary Fig. 2).

It is now possible to derive the parameter $D^2 = d^2/\sigma^2$ introduced in the main text. We defined d to be the difference between two outcomes with one and zero phonons respectively,

$$d = V_{\rm M}(n_{\rm b} = 1) - V_{\rm M}(n_{\rm b} = 0) = \alpha\sqrt{\frac{\hbar\omega_{\rm s}Z_{\rm out}}{2}}{\rm Im}\{\zeta(\omega_{\rm s}, n_{\rm b} = 0) - \zeta(\omega_{\rm s}, n_{\rm b} = 1)\}. \tag{21}$$

Considering that σ^2 is the variance $(\Delta\hat{V}_{\rm M})^2$, we find

$$D^2 = \frac{d^2}{\sigma^2} = \frac{4}{1 + 2\bar{n}_{\rm e}}\frac{g_2^2|\alpha|^2\gamma_{\rm t}^2}{\left[g_2^2 + (\gamma_{\rm t} + \gamma_{\rm r})^2\right]^2}. \tag{22}$$

This last equation reduces to the form given in the main text by setting $\gamma_{\rm r} = \gamma_{\rm t}$ and noticing that typically $g_2 \ll \gamma_{\rm r} + \gamma_{\rm t}$.

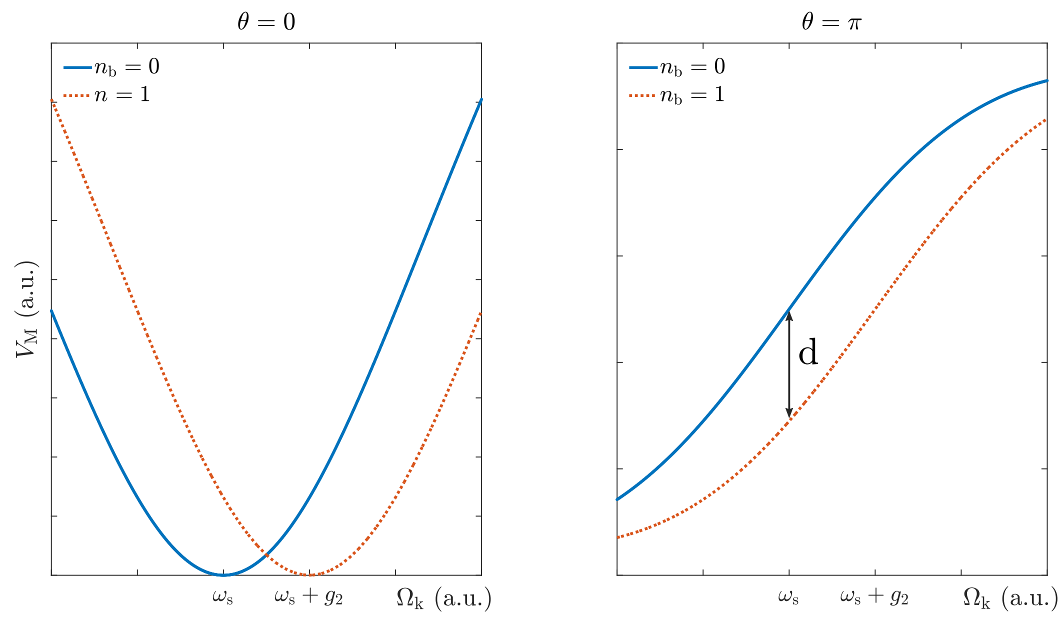

Supplementary Fig. 2: **Homodyne measurement and sensitivity**. Example of amplitude **(a)** and phase **(b)** quadratures of the measured operator $\hat{V}_{\rm M}$. The plain and dotted curves refer, respectively, to the cases in which the membrane is in its ground ($n_b = 0$) or in its first excited ($n_b = 1$) state. As one can see, the quadratic coupling g_2 induces a frequency shift, proportional to the phonon number. The quantity d, derived analytically in Supplementary Eq. (21), is indicated. We assumed optimally loaded setups with $\gamma_{\rm r} = \gamma_{\rm t}$. Compared to the realistic scenario $g_2 \ll \gamma_{\rm t}$, the frequency shift is here exaggerated. Whenever $g_2 \ll \gamma_{\rm t}$, the frequency shift is more easily detected by looking at the phase quadrature $\theta = \pi$ of the reflected signal.

Membrane heating

In this subsection we study the time evolution of the average phonon number $n_{\rm b}(t) = \langle \hat{n}_{\rm b}(t) \rangle$, and determine the parameter $\Delta n_{\rm b}$ that characterizes the probability for the mechanical state to jump during the measurement time T. It is convenient to introduce creation $\hat{a}^\dagger$ and annihilation $\hat{a}$ operators for the electrical charge $\hat{Q}$ and flux $\hat{\Phi}$ operators,

$$\hat{Q} = \sqrt{\frac{\hbar C_0 \omega_{\rm s}}{2}} (\hat{a} + \hat{a}^\dagger), \tag{23}$$

$$\hat{\Phi} = i \sqrt{\frac{\hbar}{2 C_0 \omega_{\rm s}}} (\hat{a}^\dagger - \hat{a}). \tag{24}$$

Using these we can rewrite the Hamiltonian in Supplementary Eq. (1) as

$$\hat{\mathcal{H}} = \hbar\omega_{\rm s}\hat{a}^\dagger\hat{a} + \hbar\omega_{\rm m}\hat{b}^\dagger\hat{b} + \frac{\hbar g_1}{2}\hat{a}^\dagger\hat{a}(\hat{b}+\hat{b}^\dagger) + \frac{\hbar g_2}{2}\hat{a}^\dagger\hat{a}\left(\hat{b}^\dagger\hat{b} + \frac{\hat{b}\hat{b}+\hat{b}^\dagger\hat{b}^\dagger}{2}\right) - \hbar\sqrt{\frac{2C_0\omega_{\rm s}}{\hbar}}(\hat{a}+\hat{a}^\dagger)(\hat{V}_{\rm in}+\hat{V}_{\rm R0}) - x_0\hat{F}_{\rm b}(\hat{b}+\hat{b}^\dagger), \tag{25}$$

where we have used the rotating wave approximation to neglect terms which are off–resonant with the electrical frequency $\omega_{\rm s}$. Given that the coupling coefficients g_1 and g_2 are small compared to all other parameters, we linearize $\hat{a}$ and $\hat{a}^\dagger$ such that only deviations from their steady states are considered: $\hat{a}^{(\dagger)} = \langle \hat{a}^{(\dagger)} \rangle + \hat{\delta a}^{(\dagger)}$, where

$$\langle \hat{a}(t) \rangle \xrightarrow{\rm steady} \frac{2 i \alpha \sqrt{\gamma_{\rm t}} e^{-i\omega_{\rm s} t}}{\sqrt{T}(\gamma_{\rm t} + \gamma_{\rm r})}. \tag{26}$$

If the experiment is performed in a pulsed fashion with incoming pulses varying on a timescale comparable to the circuit's lifetime $(\gamma_{\rm r}+\gamma_{\rm t})^{-1}$, Supplementary Eq. (26) should be replaced by a suitable expression that takes into account

the transient dynamics [3]. Here we restrict ourselves to the cases in which either fields are applied continuously, or pulses are slowly varying on the timescale identified by $(\gamma_\mathrm{r}+\gamma_\mathrm{t})^{-1}$. In the latter case, the simple replacement $\alpha \to \alpha(t)$ in Supplementary Eq. (26) is sufficient.

Considering that $g_2 \ll g_1$, we can neglect the quadratic coupling in the Hamiltonian Supplementary Eq. (25) for calculating the heating. The Hamiltonian can then be rewritten in the form

$$\hat{\mathcal{H}} = \hbar\omega_\mathrm{m}\hat{b}^\dagger\hat{b}+i\frac{\hbar g_1\alpha}{\gamma_\mathrm{t}+\gamma_\mathrm{r}}\sqrt{\frac{\gamma_\mathrm{t}}{T}}(\hat{\delta a}-\hat{\delta a}^\dagger)(\hat{b}+\hat{b}^\dagger)-x_0\hat{F}_\mathrm{b}(\hat{b}+\hat{b}^\dagger)-\hbar\sqrt{\frac{2C_0\omega_\mathrm{s}}{\hbar}}\left[\hat{\delta a}^\dagger\left(\delta\hat{V}_\mathrm{in}+\hat{V}_\mathrm{R0}\right)+\hat{\delta a}\left(\delta\hat{V}_\mathrm{in}^\dagger+\hat{V}_\mathrm{R0}^\dagger\right)\right], \quad (27)$$

where we have switched to the rotating frame using the unitary transformation $\hat{U} = e^{it\omega_\mathrm{s}\hat{\delta a}^\dagger\hat{\delta a}}$. Notice that we linearized the operator $\hat{V}_\mathrm{in} = \langle\hat{V}_\mathrm{in}\rangle + \delta\hat{V}_\mathrm{in}$, with its average given by the coherent field α at the frequency ω_s, and neglected a term $\propto |\alpha|^2(\hat{b}+\hat{b}^\dagger)$, that can be removed by changing the rest position of the mechanical oscillator.

The equations of motion for the operators $\hat{b}$ and $\hat{\delta a}$ are then given by

$$\dot{\hat{\delta a}} = \frac{g_1\alpha}{\gamma_\mathrm{t}+\gamma_\mathrm{r}}\sqrt{\frac{\gamma_\mathrm{t}}{T}}(\hat{b}+\hat{b}^\dagger) - \frac{\gamma_\mathrm{t}+\gamma_\mathrm{r}}{2}\hat{\delta a} + i\sqrt{\frac{2C_0\omega_\mathrm{s}}{\hbar}}(\delta\hat{V}_\mathrm{in}+\hat{V}_{R_0}), \quad (28)$$

$$\dot{\hat{b}} = -i\omega_\mathrm{m}\hat{b} - \frac{g_1\alpha}{\gamma_\mathrm{t}+\gamma_\mathrm{r}}\sqrt{\frac{\gamma_\mathrm{t}}{T}}(\hat{\delta a}-\hat{\delta a}^\dagger) - \frac{\gamma_\mathrm{b}}{2}\hat{b} + i\frac{x_0}{\hbar}\hat{F}_\mathrm{b}, \quad (29)$$

where we have linearised in the operators $\hat{\delta a}$ and $\hat{\delta a}^\dagger$. A formal solution in the time domain for these two differential operator equations then reads

$$\begin{aligned}\hat{\delta a}(t) =& \hat{\delta a}(0)e^{-\frac{\gamma_\mathrm{t}+\gamma_\mathrm{r}}{2}t} + \frac{g_1\alpha}{\gamma_\mathrm{t}+\gamma_\mathrm{r}}\sqrt{\frac{\gamma_\mathrm{t}}{T}}\int_0^\mathrm{t} e^{-\frac{\gamma_\mathrm{t}+\gamma_\mathrm{r}}{2}(t-\tau)}\left[\hat{b}(\tau)+\hat{b}^\dagger(\tau)\right]d\tau \\ &+ i\sqrt{\frac{2C_0\omega_\mathrm{s}}{\hbar}}\int_0^\mathrm{t} e^{-\frac{\gamma_\mathrm{t}+\gamma_\mathrm{r}}{2}(t-\tau)}\left[\delta\hat{V}_\mathrm{in}(\tau)+\hat{V}_{R_0}(\tau)\right]d\tau,\end{aligned} \quad (30)$$

$$\begin{aligned}\hat{b}(t) =& \hat{b}(0)e^{-\left(i\omega_\mathrm{m}+\frac{\gamma_\mathrm{b}}{2}\right)t} - \frac{g_1\alpha}{\gamma_\mathrm{t}+\gamma_\mathrm{r}}\sqrt{\frac{\gamma_\mathrm{t}}{T}}\int_0^\mathrm{t} e^{-\left(i\omega_\mathrm{m}+\frac{\gamma_\mathrm{b}}{2}\right)(t-\tau)}\left[\hat{\delta a}(\tau)-\hat{\delta a}^\dagger(\tau)\right]d\tau \\ &+ i\frac{x_0}{\hbar}\int_0^\mathrm{t} e^{-\left(i\omega_\mathrm{m}+\frac{\gamma_\mathrm{b}}{2}\right)(t-\tau)}\hat{F}_\mathrm{b}(\tau)d\tau.\end{aligned} \quad (31)$$

Since we are interested in the average phonon number $\langle\hat{b}^\dagger\hat{b}(t)\rangle$, we can substitute Supplementary Eq. (30) into Supplementary Eq. (31), to derive the time evolution of the operator $\hat{b}$ as a function of the noises $\delta\hat{V}_\mathrm{in}$, $\hat{V}_\mathrm{R0}$ and $\hat{F}_\mathrm{b}$:

$$\begin{aligned}\hat{b}(t) =& \hat{b}(0)e^{-\left(i\omega_\mathrm{m}+\frac{\gamma_\mathrm{b}}{2}\right)t} + i\frac{x_0}{\hbar}\int_0^\mathrm{t} e^{-\left(i\omega_\mathrm{m}+\frac{\gamma_\mathrm{b}}{2}\right)(t-\tau)}\hat{F}_\mathrm{b}(\tau)d\tau \\ &- \frac{2g_1\alpha\left[\hat{\delta a}(0)-\hat{\delta a}^\dagger(0)\right]}{(\gamma_\mathrm{t}+\gamma_\mathrm{r})(\gamma_\mathrm{t}+\gamma_\mathrm{r}-\gamma_\mathrm{b}-2i\omega_\mathrm{m})}\sqrt{\frac{\gamma_\mathrm{t}}{T}}\left(e^{-\frac{\gamma_\mathrm{t}+\gamma_\mathrm{r}}{2}t} - e^{-\left(i\omega_\mathrm{m}+\frac{\gamma_\mathrm{b}}{2}\right)t}\right) \\ &+ \frac{ig_1\alpha}{\gamma_\mathrm{t}+\gamma_\mathrm{r}}\sqrt{\frac{\gamma_\mathrm{t}}{T}}\sqrt{\frac{2C_0\omega_\mathrm{s}}{\hbar}}\int_0^\mathrm{t}\int_0^{\tau_1}\Bigg\{e^{-\left(i\omega_\mathrm{m}+\frac{\gamma_\mathrm{b}}{2}\right)(t-\tau_1)}e^{-\left(\frac{\gamma_\mathrm{t}+\gamma_\mathrm{r}}{2}\right)(\tau_1-\tau_2)} \\ &\times\left[\delta\hat{V}_\mathrm{in}(\tau_2)+\delta\hat{V}_\mathrm{in}^\dagger(\tau_2)+\hat{V}_\mathrm{R0}(\tau_2)+\hat{V}_\mathrm{R0}^\dagger(\tau_2)\right]\Bigg\}d\tau_1 d\tau_2.\end{aligned} \quad (32)$$

Importantly, because we choose to probe the system at the electrical resonance frequency ω_s, the oscillating terms proportional to $\hat{b}(\tau)$ and $\hat{b}^\dagger(\tau)$ in Supplementary Eq. (30) cancel each other, such that the result simplifies to Supplementary Eq. (32). The above expression is an integral operator equation, the solution of which fully describes the mechanical annihilation operator $\hat{b}(t)$. An analogous relation can be obtained for the creation operator $\hat{b}^\dagger(t)$, by taking the adjoint of Supplementary Eq. (32). In general, this integral is difficult to evaluate. However, assuming the Markov approximation, we can compute the second moments of all the noise operators involved. Thus, even if

we cannot solve Supplementary Eq. (32) for $\hat{b}(t)$ or $\hat{b}^\dagger(t)$, we can use (32) for determining $n_\mathrm{b}(t) = \langle \hat{b}^\dagger(t)\hat{b}(t)\rangle$. In particular, we use:

$$\left\langle \delta\hat{V}^\dagger_\mathrm{in}(\tau_1)\delta\hat{V}_\mathrm{in}(\tau_2)\right\rangle = \frac{\hbar\gamma_\mathrm{t}}{2C_0\omega_\mathrm{s}}\bar{n}_\mathrm{e}(\omega_\mathrm{s},T_\mathrm{e})\delta(\tau_2-\tau_1), \tag{33}$$

$$\left\langle \delta\hat{V}_\mathrm{in}(\tau_1)\delta\hat{V}^\dagger_\mathrm{in}(\tau_2)\right\rangle = \frac{\hbar\gamma_\mathrm{t}}{2C_0\omega_\mathrm{s}}\left[\bar{n}_\mathrm{e}(\omega_\mathrm{s},T_\mathrm{e})+1\right]\delta(\tau_2-\tau_1), \tag{34}$$

$$\left\langle \hat{V}^\dagger_\mathrm{R0}(\tau_1)\hat{V}_\mathrm{R0}(\tau_2)\right\rangle = \frac{\hbar\gamma_\mathrm{r}}{2C_0\omega_\mathrm{s}}\bar{n}_\mathrm{e}(\omega_\mathrm{s},T_\mathrm{e})\delta(\tau_2-\tau_1), \tag{35}$$

$$\left\langle \hat{V}_\mathrm{R0}(\tau_1)\hat{V}^\dagger_\mathrm{R0}(\tau_2)\right\rangle = \frac{\hbar\gamma_\mathrm{r}}{2C_0\omega_\mathrm{s}}\left[\bar{n}_\mathrm{e}(\omega_\mathrm{s},T_\mathrm{e})+1\right]\delta(\tau_2-\tau_1), \tag{36}$$

$$\left\langle \hat{F}^\dagger_\mathrm{b}(\tau_1)\hat{F}_\mathrm{b}(\tau_2)\right\rangle = 2\hbar m\omega_\mathrm{m}\bar{n}_\mathrm{m}(\omega_\mathrm{m},T_\mathrm{m})\delta(\tau_2-\tau_1), \tag{37}$$

$$\left\langle \hat{F}_\mathrm{b}(\tau_1)\hat{F}^\dagger_\mathrm{b}(\tau_2)\right\rangle = 2\hbar m\omega_\mathrm{m}\left[\bar{n}_\mathrm{m}(\omega_\mathrm{m},T_\mathrm{m})+1\right]\delta(\tau_2-\tau_1), \tag{38}$$

where $\bar{n}_\mathrm{e}(\omega_\mathrm{s},T_\mathrm{e})$ and $\bar{n}_\mathrm{m}(\omega_\mathrm{m},T_\mathrm{m})$ are the average thermal occupation numbers of the electrical and mechanical reservoirs at temperatures T_e and T_m, respectively. In the following, we omit their argument, assuming that they refer to the frequencies ω_s (electrical) and ω_m (mechanical), and temperatures T_e (electrical) and T_m (mechanical) of the respective systems. The requirements on the reservoirs necessary to write the second moments of the noises as in Supplementary Eqs. (33), (34), (35), (36), (37) and (38) are the following. First, the mechanical force $\hat{F}_\mathrm{b}$ needs to vary on a timescale much faster than the membrane's decay time, which is fulfilled for $\gamma_\mathrm{b} \ll \omega_\mathrm{m}$. Second, we have neglected the difference between the electrical excitation number $\bar{n}_\mathrm{e}$ at the central frequency and the mechanical sidebands. This means that the electrical resonance frequency has to be much bigger than the mechanical one ($\omega_\mathrm{s} \gg \omega_\mathrm{m}$). Both these requirements are satisfied for the considered experimental parameters.

Knowing the second moments of all noise operators, and neglecting the term proportional to $\langle \hat{\delta a}^\dagger(0)\hat{\delta a}(0)\rangle$, using Supplementary Eq. (32) we can find $n_\mathrm{b}(t) = \langle \hat{n}_\mathrm{b}(t)\rangle$ to be

$$\begin{aligned} n_\mathrm{b}(t) = {} & n_\mathrm{b}(0)e^{-\gamma_\mathrm{b}t} + \bar{n}_\mathrm{m}\left(1-e^{-\gamma_\mathrm{b}t}\right) - \frac{4g_1^2|\alpha|^2\gamma_\mathrm{t}(1+2\bar{n}_\mathrm{e})e^{-(\gamma_\mathrm{t}+\gamma_\mathrm{r})t}}{T(\gamma_\mathrm{t}+\gamma_\mathrm{r})^2\left[(-\gamma_\mathrm{b}+\gamma_\mathrm{t}+\gamma_\mathrm{r})^2+4\omega_\mathrm{m}^2\right]} \\ & + \frac{8g_1^2|\alpha|^2\gamma_\mathrm{t}(1+2\bar{n}_\mathrm{e})e^{-\frac{\gamma_\mathrm{b}+\gamma_\mathrm{t}+\gamma_\mathrm{r}}{2}t}}{T(\gamma_\mathrm{t}+\gamma_\mathrm{r})\left[(-\gamma_\mathrm{b}+\gamma_\mathrm{t}+\gamma_\mathrm{r})^2+4\omega_\mathrm{m}^2\right]}\left(\frac{e^{-i\omega_\mathrm{m}t}}{\gamma_\mathrm{b}+\gamma_\mathrm{t}+\gamma_\mathrm{r}+2i\omega_\mathrm{m}}\right. \\ & \left.+\frac{e^{i\omega_\mathrm{m}t}}{\gamma_\mathrm{b}+\gamma_\mathrm{t}+\gamma_\mathrm{r}-2i\omega_\mathrm{m}}\right) + \frac{4g_1^2|\alpha|^2\gamma_\mathrm{t}(\gamma_\mathrm{b}+\gamma_\mathrm{t}+\gamma_\mathrm{r})(1+2\bar{n}_\mathrm{e})}{T\gamma_\mathrm{b}(\gamma_\mathrm{t}+\gamma_\mathrm{r})^2\left[(-\gamma_\mathrm{b}+\gamma_\mathrm{t}+\gamma_\mathrm{r})^2+4\omega_\mathrm{m}^2\right]} \\ & - \frac{4g_1^2|\alpha|^2\gamma_\mathrm{t}(1+2\bar{n}_\mathrm{e})e^{-\gamma_\mathrm{b}t}}{T\gamma_\mathrm{b}(\gamma_\mathrm{t}+\gamma_\mathrm{r})\left[(-\gamma_\mathrm{b}+\gamma_\mathrm{t}+\gamma_\mathrm{r})^2+4\omega_\mathrm{m}^2\right]}, \end{aligned} \tag{39}$$

where the first two terms $n_\mathrm{b}(0)e^{-\gamma_\mathrm{b}t} + \bar{n}_\mathrm{m}\left(1-e^{-\gamma_\mathrm{b}t}\right)$ describe the usual time evolution of a free membrane influenced by its own thermal bath, and everything else is the dynamics induced from the electrical system.

Supplementary Eq. (39) represents an exact result in the limit in which the photonic ($\bar{n}_\mathrm{e}$) and phononic ($\bar{n}_\mathrm{m}$) reservoirs are Markovian. However, it can be better understood if we assume the mechanical damping γ_b to be much smaller than the electrical one, $\gamma_\mathrm{t}+\gamma_\mathrm{r}$. In particular, if we are probing the system on a time scale that is much longer than the electrical lifetime, i.e. $t \gg (\gamma_\mathrm{t}+\gamma_\mathrm{r})^{-1}$, we can rewrite Supplementary Eq. (39) as

$$n_\mathrm{b}(t) = n_\mathrm{b}(0)e^{-\gamma_\mathrm{b}t} + \left[\bar{n}_\mathrm{m} + \frac{\Gamma_\mathrm{b}}{\gamma_\mathrm{b}}(1+2\bar{n}_\mathrm{e})\right]\left(1-e^{-\gamma_\mathrm{b}t}\right), \tag{40}$$

where we have defined the induced heating Γ_b to be

$$\Gamma_\mathrm{b} = \frac{4g_1^2|\alpha|^2\gamma_\mathrm{t}}{T(\gamma_\mathrm{t}+\gamma_\mathrm{r})\left[(\gamma_\mathrm{t}+\gamma_\mathrm{r})^2+4\omega_\mathrm{m}^2\right]}. \tag{41}$$

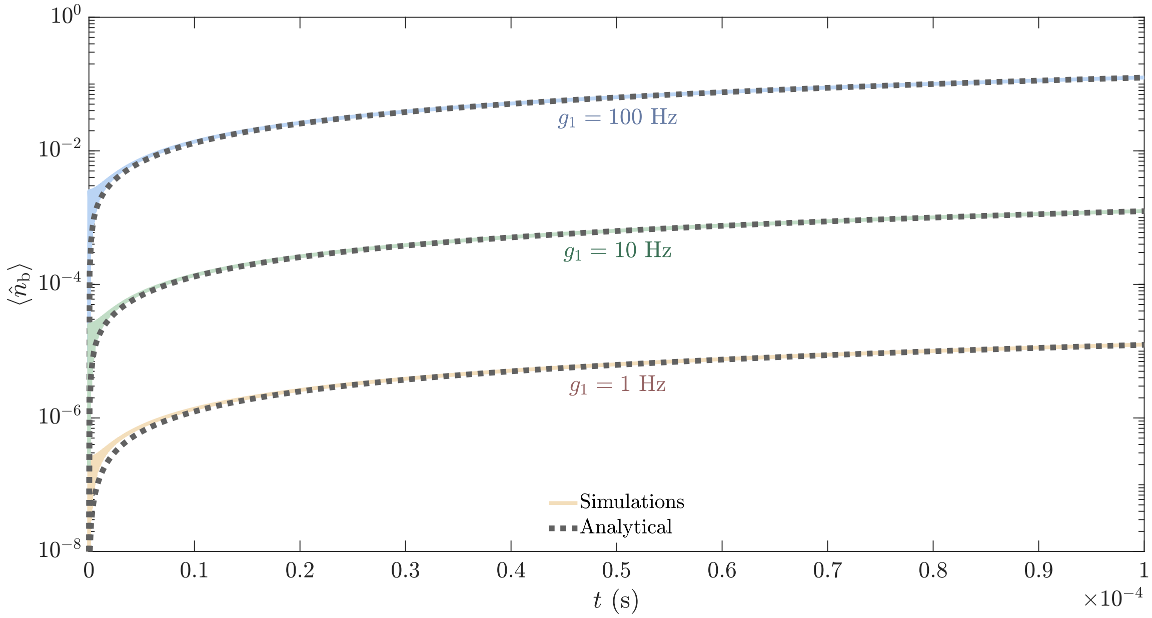

Supplementary Fig. 3: **Thermalization of the single arm circuit**. Examples for the dynamics of the average mechanical phonon number $n_b(t)$. The dotted curves are the analytical results given in Supplementary Eq. (40). The solid curves comes from numerically solving the master equation with the Hamiltonian of Supplementary Eq. (27) and the electrical and mechanical reservoirs (using the Markov approximation). The three sets differ from the parameter g_1, that has been taken to be equal to $(2\pi)100$ Hz (top set), $(2\pi)10$ Hz (middle set), and $(2\pi)1$ Hz (bottom set). We assume that $|\alpha|^2 = 10^{12}$ photons are sent into the system for the duration T of the measurement, and we used the parameters: $\omega_m = (2\pi)100$ MHz, $\omega_s = (2\pi)5$ GHz, $\gamma_r = \gamma_t = (2\pi)1$ MHz, $\gamma_b = (2\pi)100$ Hz, $T = 100\ \mu$s, $\bar{n}_e = 0$ and $\bar{n}_m = 0$.

Supplementary equation (40) has been compared with numerical simulations of the master equation of the Hamiltonian in Supplementary Eq. (27), taking into account the electrical and mechanical reservoirs. Examples are given in supplementary Fig. 3.

From the above expressions, we can determine the parameter Δn_b by finding the first order expansion of the function $n_b(t)$ in Supplementary Eq. (40) with $n_b(0) = 0$,

$$\Delta n_b = \gamma_b \bar{n}_m T + \Gamma_b (1 + 2\bar{n}_e) T. \tag{42}$$

The form of Δn_b given in the main text assumes $\gamma_b \ll \frac{\Gamma_b}{\bar{n}_m}$. Having determined both D^2 and Δn_b, it is possible to derive the general form of the parameter λ, including the contribution from the mechanical reservoir.

SUPPLEMENTARY NOTE 2

Double arm circuit

In the following we derive the parameter λ for the double arm circuit, introduced to take into account the coupling to the antisymmetric mode associated with the redistribution of charge on the membrane. This is depicted in supplementary Fig. 4. We include possible asymmetries in the fabrication process, the effect of which can be described by a residual linear coupling g_r. The contribution to λ from other kinds of asymmetries – different parasitic resistances and inductances – will only be investigated numerically, since the analytical results are too long and complicated in this case.

Using the currents $\hat{I}_s$ and $\hat{I}_a$ and their associated charges $\hat{Q}_s$ and $\hat{Q}_a$, we can determine the Hamiltonian of the

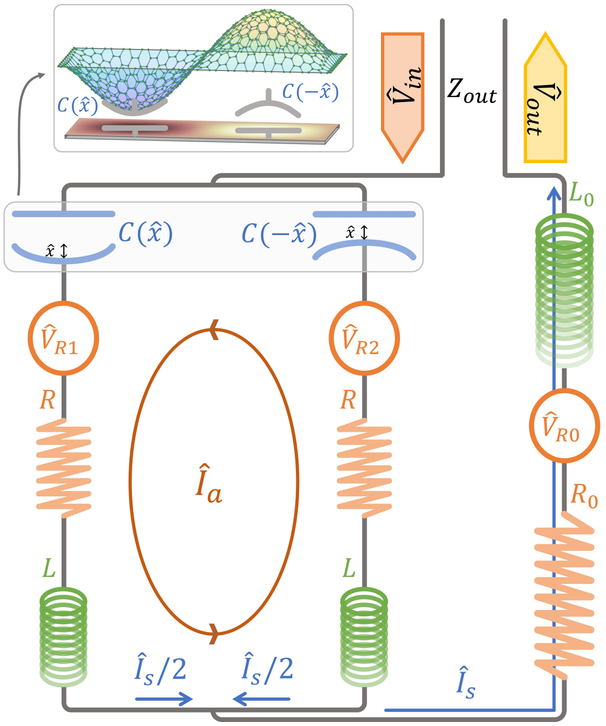

Supplementary Fig. 4: **Double arm circuit**. Double arm model of the considered electromechanical setup, with parasitic resistances (R) and inductances (L). We take into account the Johnson–Nyquist noises associated with the main resistor R_0 and the two parasitic ones: $\hat{V}_{\mathrm{R0}}$, $\hat{V}_{\mathrm{R1}}$ and $\hat{V}_{\mathrm{R2}}$, respectively. $\hat{I}_{\mathrm{s}}$ and $\hat{I}_{\mathrm{a}}$ are the two electrical currents considered in our analysis, and $C(\pm\hat{x})$ represent the two halves of the capacitor. In the inset we sketch how $C(\pm\hat{x})$ may arise.

circuit in supplementary Fig. 4 to be

$$\begin{aligned}\hat{\mathcal{H}} =&\hbar\omega_{\mathrm{m}}\hat{b}^\dagger\hat{b} - x_0\left(\hat{b}^\dagger+\hat{b}\right)\hat{F}_{\mathrm{b}} + \frac{\hat{\Phi}_{\mathrm{a}}^2}{4L} + \frac{\hat{Q}_{\mathrm{a}}^2}{C_0} + \frac{\hat{\Phi}_{\mathrm{s}}^2}{L+2L_0} + \frac{\hat{Q}_{\mathrm{s}}^2}{4C_0} - \hat{Q}_{\mathrm{s}}(2\hat{V}_{\mathrm{in}} + 2\hat{V}_{\mathrm{R0}} + \hat{V}_{\mathrm{R1}} + \hat{V}_{\mathrm{R2}}) \\ &- 2\hat{Q}_{\mathrm{a}}(\hat{V}_{\mathrm{R2}} - \hat{V}_{\mathrm{R1}}) + \frac{g_1}{C_0\omega_{\mathrm{s}}}\hat{Q}_{\mathrm{a}}\hat{Q}_{\mathrm{s}}\left(\hat{b}+\hat{b}^\dagger\right) + \frac{g_2}{C_0\omega_{\mathrm{s}}}\hat{Q}_{\mathrm{a}}^2\hat{b}^\dagger\hat{b} + \frac{g_2}{4C_0\omega_{\mathrm{s}}}\hat{Q}_{\mathrm{s}}^2\hat{b}^\dagger\hat{b},\end{aligned} \tag{43}$$

where the magnetic fluxes $\hat{\Phi}_{\mathrm{a}}$ and $\hat{\Phi}_{\mathrm{s}}$ are defined below. In principle, there are other terms proportional to $g_2\hat{b}\hat{b}$ and $g_2\hat{b}^\dagger\hat{b}^\dagger$ in the Hamiltonian. These are responsible for sidebands at frequencies $\omega_{\mathrm{s}} \pm 2\omega_{\mathrm{m}}$, that we have already encountered in the Supplementary Note 1. For the same reasons explained there, these terms do not contribute neither to the electrical readout (homodyne measurement at frequency ω_{s}) nor to the heating ($g_2 \ll g_1$), and we shall therefore ignore them in the following. A quantitative reason for neglecting these two–phonon processes, is the following. With Fermi Golden rule, we can determine the rate at which these processes happen to be

$$\frac{g_2^2|\alpha|^2\gamma_{\mathrm{t}}}{\left(\frac{\gamma_{\mathrm{r}}+\gamma_{\mathrm{t}}}{2}\right)^2 + (2\omega_{\mathrm{m}})^2}, \tag{44}$$

that is orders of magnitude lower than $\Delta n_{\mathrm{b}}/T$ (the one–phonon processes induced by the linear coupling), as can be estimated later in the Supplementary Note 4, where parameters for a proposed implementation are given. Notice that the rate in Supplementary Eq. (44) describes the two phonon processes relative to the term $\propto g_2\hat{Q}_{\mathrm{s}}^2$ in the Hamiltonian in Supplementary Eq. (43). The other two phonon process, $\propto g_2\hat{Q}_{\mathrm{a}}^2$, is even more suppressed, as the asymmetric field is not directly driven by the input $\hat{V}_{\mathrm{in}}$.

From Supplementary Eq. (43), it is possible to derive the equations of motion for the fields $\hat{Q}_{\mathrm{a}}$, $\hat{Q}_{\mathrm{s}}$, $\hat{\Phi}_{\mathrm{a}}$, $\hat{\Phi}_{\mathrm{s}}$ and $\hat{b}$. The electrical decays are included using Kirchoff laws, resulting in:

$$\dot{\hat{\Phi}}_{\mathrm{a}} = -2\frac{\hat{Q}_{\mathrm{a}}}{C_0} - \frac{g_1}{C_0\omega_{\mathrm{s}}}\hat{Q}_{\mathrm{s}}\left(\hat{b}+\hat{b}^\dagger\right) - \frac{2g_2}{C_0\omega_{\mathrm{s}}}\hat{Q}_{\mathrm{a}}\hat{n}_{\mathrm{b}} - \gamma_l\hat{\Phi}_{\mathrm{a}} + 2\left(\hat{V}_{\mathrm{R2}} - \hat{V}_{\mathrm{R1}}\right), \tag{45}$$

$$\dot{\hat{Q}}_{\rm a} = \frac{\hat{\Phi}_{\rm a}}{2L}, \tag{46}$$

$$\dot{\hat{\Phi}}_{\rm s} = -\frac{\hat{Q}_{\rm s}}{2C_0} - \frac{g_1}{C_0\omega_{\rm s}}\hat{Q}_{\rm a}\left(\hat{b}+\hat{b}^\dagger\right) - \frac{g_2}{2C_0\omega_{\rm s}}\hat{Q}_{\rm s}\hat{n}_{\rm b} - (\gamma_{\rm t}+\gamma_{\rm r})\hat{\Phi}_{\rm s} + 2\hat{V}_{\rm in} + 2\hat{V}_{\rm R0} + \hat{V}_{\rm R1} + \hat{V}_{\rm R2}, \tag{47}$$

$$\dot{\hat{Q}}_{\rm s} = \frac{2\hat{\Phi}_{\rm s}}{L+2L_0}, \tag{48}$$

$$\dot{\hat{b}} = -i\omega_{\rm m}\hat{b} - \frac{i}{\hbar}\frac{g_1}{C_0\omega_{\rm s}}\hat{Q}_{\rm a}\hat{Q}_{\rm s} - \frac{i}{\hbar}\frac{g_2}{C_0\omega_{\rm s}}\left(\hat{Q}_{\rm a}^2 + \frac{\hat{Q}_{\rm s}^2}{4}\right)\hat{b} - \frac{\gamma_{\rm b}}{2}\hat{b} + \frac{x_0}{\hbar}\hat{F}_{\rm b}. \tag{49}$$

To enable a direct comparison with the RLC circuit presented in the Supplementary Note 1 we have here used the same notation. This requires small differences in the definitions, to take into account the parasitic resistances and inductances:

$$\omega_{\rm s}^2 = \frac{1}{C_0(L+2L_0)}, \tag{50}$$

$$\omega_{\rm a}^2 = \frac{1}{C_0 L}, \tag{51}$$

$$\gamma_{\rm t} = \frac{2Z_{\rm out}}{L+2L_0}, \tag{52}$$

$$\gamma_{\rm r} = \frac{R+2R_0}{L+2L_0}, \tag{53}$$

$$\gamma_l = \frac{R}{L}. \tag{54}$$

Moreover, the linear and quadratic couplings g_1 and g_2 come from the expansions of each one of the two capacitors $C(\pm\hat{x})$ in the mechanical position $\hat{x}$, as suggested by the inset in supplementary Fig. 4. Following the same procedure used for the RLC circuit, we first study the measurement of $\hat{n}_{\rm b}$, and later identify the conditions under which the measurement is effectively QND.

Notice that, depending on the specific experimental setup, other circuit scheme may be better suited for describing the system. For instance, in the experiment of Ref. [4] the mechanical oscillator is not directly connected to the circuit, and different equivalent circuit would be required. Here, we restrict ourselves to the setup in supplementary Fig. 4 and defer other setup for later investigation [5].

QND measurement of the phonon number, and comparison with the membrane in the middle setup [6]

Assuming that the mechanical state is unchanged during the whole measurement time T, we can neglect all sources of heating in the system of equations (45), (46), (47), (48), (49), and take $\hat{n}_{\rm b}$ to be constant in time. Importantly, for the setup in supplementary Fig. 4 there are two mechanisms that shift the resonant frequency $\omega_{\rm s}$ and thus allow for the QND measurement. The first one we encountered before for the RLC circuit, and relates to the quadratic electromechanical coupling: $\omega_{\rm s} \to \omega_{\rm s} + g_2 n_{\rm b}$. The second is more involved, and is the same one considered in the optomechanical setup of Ref. [7]. It relies on an effective quadratic coupling proportional to g_1^2, that arises once we substitute Supplementary Eqs. (45) and (46) into Supplementary Eqs. (47) and (48). To better understand this process, it is instructive to look at the final equation for $\hat{\Phi}_{\rm s}$ in the frequency domain:

$$\left(\Omega_{\rm k}^2 - \omega_{\rm s}^2 + \overbrace{\frac{(\hat{n}_{\rm b}+1)g_1^2\omega_{\rm a}^2}{\omega_{\rm a}^2 + (\Omega_{\rm k}-\omega_{\rm m})(-i\gamma_l - \Omega_{\rm k} + \omega_{\rm m})} + \frac{\hat{n}_{\rm b} g_1^2\omega_{\rm a}^2}{\omega_{\rm a}^2 - (\Omega_{\rm k}+\omega_{\rm m})(i\gamma_l + \Omega_{\rm k} + \omega_{\rm m})}}^{g_1^2\text{ shift}} - \overbrace{g_2\omega_{\rm s}\hat{n}_{\rm b}}^{g_2\text{ shift}}\right)\hat{\Phi}_{\rm s}[\Omega_{\rm k}] = \tag{55}$$
$$i\Omega_{\rm k}(\gamma_{\rm r}+\gamma_{\rm t})\hat{\Phi}_{\rm s}[\Omega_{\rm k}] + 2i\Omega_{\rm k}\hat{V}_{\rm in}[\Omega_{\rm k}] + 2i\Omega_{\rm k}\hat{V}_{\rm R0}[\Omega_{\rm k}] + i\Omega_{\rm k}\hat{V}_{\rm R1}[\Omega_{\rm k}] + i\Omega_{\rm k}\hat{V}_{\rm R2}[\Omega_{\rm k}],$$

where we neglected off–resonant terms and assumed $g_2 \ll \omega_{\rm s}$. As indicated explicitly in Supplementary Eq. (55), it is possible to see the frequency shifts induced by the linear g_1 and quadratic g_2 couplings. Whether the QND interaction is dominated by the quadratic coupling g_2 or the effective quadratic interaction $\propto g_1^2$, depends on the resonance condition of the symmetric $\hat{I}_{\rm s}$ and antisymmetric $\hat{I}_{\rm a}$ modes in supplementary Fig. 4. To have a sizeable effect of the g_1 term, we need to be near resonance with the antisymmetric mode $\Omega_{\rm k} \simeq \omega_{\rm a} \gg \omega_{\rm m}, \gamma_l$. At the same time, for the QND detection we probe the system at the resonance frequency $\Omega_{\rm k} \simeq \omega_{\rm s}$. Hence, the g_1 term is dominant when we allow for a strong hybridization of the two electrical modes: $\omega_{\rm s} \simeq \omega_{\rm a}$. In such situation, by determining the heating rate for the electromechanical setup (see Supplementary Note 1), we can derive the condition for a feasible QND detection:

$$\lambda = \frac{2}{(1+\bar{n}_{\rm e})(1+2\bar{n}_{\rm e})} \frac{g_1^2}{(\gamma_{\rm r}+\gamma_{\rm t})^2} \frac{\gamma_{\rm t}}{\gamma_l} \gg 1. \tag{56}$$

This condition is similar to the one found in Ref. [6] for the experiment in Ref. [7], where strong heating was proven to forbid the QND detection. Compared to that work, however, we gain the factor $\gamma_{\rm t}/\gamma_l$, that comes from the asymmetry between the damping of the two electrical modes. Hence, our setup does have some gain compared to the RLC circuit, if the damping of the antisymmetric mode is small: $\gamma_l \ll \gamma_{\rm t}$. However, in this case the QND measurement still faces the challenge of a strong heating of the mechanical motion due to the large coupling between the electrical modes, and requires $g_1^2 \gg \gamma_l \gamma_{\rm t}$.

In the following, we will investigate the opposite limit, where the antisymmetric mode $\hat{I}_{\rm a}$ is far–off resonant from the symmetric one $\hat{I}_{\rm s}$. In this case, the quadratic coupling g_2 is dominant (if $g_2 \simeq g_1^2/\omega_s$, one can simply redefine the quadratic coupling to take all contributions into account), and the heating induced to the mechanical mode will be strongly suppressed. If we neglect the effective quadratic coupling $\propto g_1^2$, we only need to consider Supplementary Eqs. (47) and (48) for determining the measurement signal. In the absence of heating, these are uncoupled from both the mechanics and the other electrical modes. We can then derive relations for $\hat{\Phi}_{\rm s}[\Omega_{\rm k}]$ and $\hat{Q}_{\rm s}[\Omega_{\rm k}]$ similar to Supplementary Eqs. (11) and (12):

$$-i\Omega_{\rm k}\hat{\Phi}_{\rm s}[\Omega_{\rm k}] = -\frac{\hat{Q}_{\rm s}[\Omega_{\rm k}]}{2C_0} - \frac{g_2\omega_{\rm s}L_0}{2}\hat{Q}_{\rm s}[\Omega_{\rm k}]\hat{n}_{\rm b} - (\gamma_{\rm r}+\gamma_{\rm t})\hat{\Phi}_{\rm s}[\Omega_{\rm k}] + 2(\hat{V}_{\rm in}[\Omega_{\rm k}] + \hat{V}_{\rm R0}[\Omega_{\rm k}]) + \hat{V}_{\rm R1}[\Omega_{\rm k}] + \hat{V}_{\rm R2}[\Omega_{\rm k}], \tag{57}$$

$$-i\Omega_{\rm k}\hat{Q}_{\rm s}[\Omega_{\rm k}] = \frac{2\hat{\Phi}_{\rm s}[\Omega_{\rm k}]}{L+2L_0}. \tag{58}$$

The reflected field $\hat{V}_{\rm out}[\Omega_{\rm k}]$ is

$$\hat{V}_{\rm out}[\Omega_{\rm k}] = [1-\zeta(\Omega_{\rm k},\hat{n}_{\rm b})]\,\hat{V}_{\rm in}[\Omega_{\rm k}] - \zeta(\Omega_{\rm k},\hat{n}_{\rm b})\left(\hat{V}_{\rm R0}[\Omega_{\rm k}] + \frac{\hat{V}_{\rm R1}[\Omega_{\rm k}]+\hat{V}_{\rm R2}[\Omega_{\rm k}]}{2}\right), \tag{59}$$

where we have used the input/output relation $\hat{V}_{\rm out} = \hat{V}_{\rm in} - \gamma_{\rm t}\hat{\Phi}_{\rm s}$. The coefficient $\zeta(\Omega_{\rm k},\hat{n}_{\rm b})$ is the same as in Supplementary Eq. (14), except for a factor 2 coming from the fact that, here, we are considering the two halves of our capacitor. With the outcome of the homodyne detection being described by the same operator $\hat{V}_{\rm M}$ defined in Supplementary Eq. (17), we can find the parameter D^2 for the setup in supplementary Fig. 4 to be

$$D^2 = \frac{d^2}{\sigma^2} = \frac{16}{1+2\bar{n}_{\rm e}} \frac{g_2^2|\alpha|^2\gamma_{\rm t}^2}{\left[g_2^2+(\gamma_{\rm t}+\gamma_{\rm r})^2\right]^2}. \tag{60}$$

Notice that for deriving Supplementary Eq. (60) from Supplementary Eq. (59) we assumed that the electrical reservoirs are in a thermal state with average photon number $\bar{n}_{\rm e}$, and that the drive is a coherent state α at the frequency $\omega_{\rm s}$. Consistent with previous sections, α is chosen to be real and the phase of the homodyne measurement is fixed such that $\theta = \pi$ in Supplementary Eq. (17).

Membrane heating

Given that $g_2 \ll g_1$, we set the quadratic coupling to zero in this subsection. We can then rewrite the Hamiltonian in Supplementary Eq. (43) in the form

$$\begin{aligned}\hat{\mathcal{H}} = &\hbar\omega_\mathrm{m}\hat{b}^\dagger\hat{b} - x_0\left(\hat{b}^\dagger+\hat{b}\right)\hat{F}_\mathrm{b} + \frac{\hat{\Phi}_\mathrm{a}^2}{4L} + \frac{\hat{Q}_\mathrm{a}^2}{C_0} + \frac{\hat{\Phi}_\mathrm{s}^2}{L+2L_0} + \frac{\hat{Q}_\mathrm{s}^2}{4C_0} - \hat{Q}_\mathrm{s}(2\hat{V}_\mathrm{in} + 2\hat{V}_\mathrm{R0} + \hat{V}_\mathrm{R1} + \hat{V}_\mathrm{R2}) \\ &- 2\hat{Q}_\mathrm{a}(\hat{V}_\mathrm{R2} - \hat{V}_\mathrm{R1}) + \frac{g_1}{C_0\omega_\mathrm{s}}\hat{Q}_\mathrm{a}\hat{Q}_\mathrm{s}\left(\hat{b}+\hat{b}^\dagger\right).\end{aligned} \tag{61}$$

Moreover, by looking at Supplementary Eqs. (45), (46), (47), (48), (49), it is possible to see that $\hat{Q}_\mathrm{s}$ and $\hat{\Phi}_\mathrm{s}$ are the only driven fields in the system (with $\hat{V}_\mathrm{R0}$, $\hat{V}_\mathrm{R1}$ and $\hat{V}_\mathrm{R2}$ being in a thermal state). Therefore, we can neglect perturbations induced by the mechanical motion, and substitute them with their average values $\langle\hat{Q}_\mathrm{s}\rangle$ and $\langle\hat{\Phi}_\mathrm{s}\rangle$. This assumption will be verified in later, where we simulate the dynamics of the electromechanical system in the general case in which the parasitic elements may differ from each other. From the Hamiltonian Supplementary Eq. (61) we can determine the equations of motion for $\langle\hat{Q}_\mathrm{s}\rangle$ and $\langle\hat{\Phi}_\mathrm{s}\rangle$:

$$\langle\dot{\hat{\Phi}}_\mathrm{s}(t)\rangle = -\frac{\langle\hat{Q}_\mathrm{s}(t)\rangle}{2C_0} - (\gamma_\mathrm{t}+\gamma_\mathrm{r})\langle\hat{\Phi}_\mathrm{s}(t)\rangle + 2\langle\hat{V}_\mathrm{in}(t)\rangle, \tag{62}$$

$$\langle\dot{\hat{Q}}_\mathrm{s}(t)\rangle = \frac{2\langle\hat{\Phi}_\mathrm{s}(t)\rangle}{L+2L_0}, \tag{63}$$

where $\langle\hat{V}_\mathrm{in}(t)\rangle = \alpha\sqrt{2\hbar\omega_\mathrm{s}Z_\mathrm{out}/T}\cos(\omega_\mathrm{s}t)$. Similar to above, we are mainly interested in the case of constant incident fields or long pulses, such that we ignore transient behaviours. The steady state solution for the charge $\langle\hat{Q}_\mathrm{s}(t)\rangle$ is then given by

$$\langle\hat{Q}_\mathrm{s}(t)\rangle = -\frac{2i\alpha}{\gamma_\mathrm{t}+\gamma_\mathrm{r}}\sqrt{\frac{\hbar C_0\omega_\mathrm{s}\gamma_\mathrm{t}}{T}}\left(e^{i\omega_\mathrm{s}t} - e^{-i\omega_\mathrm{s}t}\right). \tag{64}$$

Assuming that the strongly driven symmetric fields $\hat{Q}_\mathrm{s}$ and $\hat{\Phi}_\mathrm{s}$ are not perturbed by the mechanical dynamics, we can substitute Supplementary Eq. (64) into the Hamiltonian of Supplementary Eq. (61), and obtain

$$\hat{\mathcal{H}} = \hbar\omega_\mathrm{m}\hat{b}^\dagger\hat{b} - x_0\left(\hat{b}^\dagger+\hat{b}\right)\hat{F}_\mathrm{b} + \frac{\hat{\Phi}_\mathrm{a}^2}{4L} + \frac{\hat{Q}_\mathrm{a}^2}{C_0} - 2\hat{Q}_\mathrm{a}(\hat{V}_\mathrm{R1} - \hat{V}_\mathrm{R1}) - \frac{2i\hbar g_1\alpha}{\gamma_\mathrm{t}+\gamma_\mathrm{r}}\sqrt{\frac{\gamma_\mathrm{t}}{\hbar TC_0\omega_\mathrm{s}}}\left(e^{i\omega_\mathrm{s}t} - e^{-i\omega_\mathrm{s}t}\right)\hat{Q}_\mathrm{a}\left(\hat{b}+\hat{b}^\dagger\right). \tag{65}$$

Differential equations for $\hat{Q}_\mathrm{a}$, $\hat{\Phi}_\mathrm{a}$, and the mechanical annihilation operator $\hat{b}$ can finally be derived:

$$\dot{\hat{\Phi}}_\mathrm{a} = -2\frac{\hat{Q}_\mathrm{a}}{C_0} + \frac{2ig_1\alpha}{\gamma_\mathrm{t}+\gamma_\mathrm{r}}\sqrt{\frac{\hbar\gamma_\mathrm{t}}{TC_0\omega_\mathrm{s}}}\left(e^{-i\omega_\mathrm{s}t} - e^{i\omega_\mathrm{s}t}\right)\left(\hat{b}+\hat{b}^\dagger\right) - \gamma_l\hat{\Phi}_\mathrm{a} + 2\left(\hat{V}_\mathrm{R2} - \hat{V}_\mathrm{R1}\right), \tag{66}$$

$$\dot{\hat{Q}}_\mathrm{a} = \frac{\hat{\Phi}_\mathrm{a}}{2L}, \tag{67}$$

$$\dot{\hat{b}} = -i\omega_\mathrm{m}\hat{b} - \frac{2g_1\alpha}{\gamma_\mathrm{t}+\gamma_\mathrm{r}}\sqrt{\frac{\gamma_\mathrm{t}}{\hbar TC_0\omega_\mathrm{s}}}\left(e^{-i\omega_\mathrm{s}t} - e^{i\omega_\mathrm{s}t}\right)\hat{Q}_\mathrm{a} - \frac{\gamma_\mathrm{b}}{2}\hat{b} + i\frac{x_0}{\hbar}\hat{F}_\mathrm{b}. \tag{68}$$

These equations describe the coupling of the mechanical mode to the antisymmetric electrical one. This coupling is enhanced by driving the symmetric mode with the coherent state α, and is responsible for heating up the membrane, similarly to Ref. [6]. Therefore, we need to assess to which degree this is deleterious for the QND measurement of $\hat{n}_\mathrm{b}$. Supplementary Equations (66), (67) and (68) are nontrivial, and exact solutions are not accessible. However, under reasonable assumptions, we are able to find an approximate analytical solution that will be subsequently confirmed by our numerical approach.

Our first step for dealing with the system (66), (67) and (68) is to switch to the Fourier domain:

$$
\begin{aligned}
-i\Omega_{\rm k}\hat{\Phi}_{\rm a}\left[\Omega_{\rm k}\right] = & -2\frac{\hat{Q}_{\rm a}\left[\Omega_{\rm k}\right]}{C_0} + \frac{2ig_1\alpha}{\gamma_{\rm t}+\gamma_{\rm r}}\sqrt{\frac{\hbar\gamma_{\rm t}}{TC_0\omega_{\rm s}}}\Big(\hat{b}\left[\Omega_{\rm k}-\omega_{\rm s}\right] + \hat{b}^{\dagger}\left[\Omega_{\rm k}-\omega_{\rm s}\right] \\
& -\hat{b}\left[\Omega_{\rm k}+\omega_{\rm s}\right] - \hat{b}^{\dagger}\left[\Omega_{\rm k}+\omega_{\rm s}\right]\Big) - \gamma_l\hat{\Phi}_{\rm a}\left[\Omega_{\rm k}\right] + 2\left(\hat{V}_{\rm R2}\left[\Omega_{\rm k}\right] - \hat{V}_{\rm R1}\left[\Omega_{\rm k}\right]\right),
\end{aligned}
\tag{69}
$$

$$
-i\Omega_{\rm k}\hat{Q}_{\rm a}\left[\Omega_{\rm k}\right] = \frac{\hat{\Phi}_{\rm a}\left[\Omega_{\rm k}\right]}{2L},
\tag{70}
$$

$$
\begin{aligned}
-i\Omega_{\rm k}\hat{b}\left[\Omega_{\rm k}\right] = & -i\omega_{\rm m}\hat{b}\left[\Omega_{\rm k}\right] - \frac{2g_1\alpha}{\gamma_{\rm t}+\gamma_{\rm r}}\sqrt{\frac{\gamma_{\rm t}}{\hbar TC_0\omega_{\rm s}}}\left(\hat{Q}_{\rm a}\left[\Omega_{\rm k}-\omega_{\rm s}\right] - \hat{Q}_{\rm a}\left[\Omega_{\rm k}+\omega_{\rm s}\right]\right) \\
& -\frac{\gamma_{\rm b}}{2}\hat{b}\left[\Omega_{\rm k}\right] + i\frac{x_0}{\hbar}\hat{F}_{\rm b}\left[\Omega_{\rm k}\right].
\end{aligned}
\tag{71}
$$

We can employ equations (69) and (70) in order to obtain an expression for $\hat{b}\left[\Omega_{\rm k}\right]$ that depends on the noises only

$$
\begin{aligned}
\hat{b}\left[\Omega_{\rm k}\right]\left(\frac{\gamma_{\rm b}+\Gamma_{\rm b}(\Omega_{\rm k})}{2} - i\left[\Omega_{\rm k}-\omega_{\rm m}-\omega_{\rm b}(\Omega_{\rm k})\right]\right) = i\frac{x_0}{\hbar}\hat{F}_{\rm b}\left[\Omega_{\rm k}\right] + \frac{2g_1\alpha}{\gamma_{\rm t}+\gamma_{\rm r}}\omega_{\rm a}^2\sqrt{\frac{\gamma_{\rm t}C_0}{\hbar T\omega_{\rm s}}}\times \\
\times\left(\frac{\hat{V}_{\rm R2}\left[\Omega_{\rm k}-\omega_{\rm s}\right]-\hat{V}_{\rm R1}\left[\Omega_{\rm k}-\omega_{\rm s}\right]}{-\omega_{\rm a}^2+\left(\Omega_{\rm k}-\omega_{\rm s}\right)\left(i\gamma_l+\Omega_{\rm k}-\omega_{\rm s}\right)} - \frac{\hat{V}_{\rm R2}\left[\Omega_{\rm k}+\omega_{\rm s}\right]-\hat{V}_{\rm R1}\left[\Omega_{\rm k}+\omega_{\rm s}\right]}{-\omega_{\rm a}^2+\left(\Omega_{\rm k}+\omega_{\rm s}\right)\left(i\gamma_l+\Omega_{\rm k}+\omega_{\rm s}\right)}\right),
\end{aligned}
\tag{72}
$$

where we have defined the effective decay $\Gamma_{\rm b}(\Omega_{\rm k})$ and frequency shift $\omega_{\rm b}(\Omega_{\rm k})$, resulting from the electrical influence on the mechanical motion:

$$
\Gamma_{\rm b}(\Omega_{\rm k}) = {\rm Re}\left\{\frac{-4i\gamma_{\rm t}}{T\omega_{\rm s}}\left(\frac{\alpha g_1\omega_{\rm a}}{\gamma_{\rm r}+\gamma_{\rm t}}\right)^2\left(\frac{1}{-\omega_{\rm a}^2+(\Omega_{\rm k}-\omega_{\rm s})(i\gamma_l+\Omega_{\rm k}-\omega_{\rm s})} + \frac{1}{-\omega_{\rm a}^2+(\Omega_{\rm k}+\omega_{\rm s})(i\gamma_l+\Omega_{\rm k}+\omega_{\rm s})}\right)\right\},
\tag{73}
$$

$$
\omega_{\rm b}(\Omega_{\rm k}) = {\rm Im}\left\{\frac{-2i\gamma_{\rm t}}{T\omega_{\rm s}}\left(\frac{\alpha g_1\omega_{\rm a}}{\gamma_{\rm r}+\gamma_{\rm t}}\right)^2\left(\frac{1}{-\omega_{\rm a}^2+(\Omega_{\rm k}-\omega_{\rm s})(i\gamma_l+\Omega_{\rm k}-\omega_{\rm s})} + \frac{1}{-\omega_{\rm a}^2+(\Omega_{\rm k}+\omega_{\rm s})(i\gamma_l+\Omega_{\rm k}+\omega_{\rm s})}\right)\right\}.
\tag{74}
$$

Note that, for deriving Supplementary Eq. (72), we neglected the off resonant terms $\hat{b}[\Omega_{\rm k}\pm 2\omega_{\rm s}]$, $\hat{b}^{\dagger}[\Omega_{\rm k}\pm 2\omega_{\rm s}]$, and $\hat{b}^{\dagger}[\Omega_{\rm k}]$ (we verify this approximation numerically below in supplementary Fig. 6). Moreover, the Fourier components of the noises are written in their most general form, meaning that $\Omega_{\rm k}$ is now allowed to be negative, with the additional constraints $\hat{V}_{\rm R1}[\Omega_{\rm k}] = \hat{V}^{\dagger}_{\rm R1}[-\Omega_{\rm k}]$, and $\hat{V}_{\rm R2}[\Omega_{\rm k}] = \hat{V}^{\dagger}_{\rm R2}[-\Omega_{\rm k}]$.

In order to have an analytical expression for $n_{\rm b}(t) \equiv \langle\hat{n}_{\rm b}(t)\rangle$, we assume that the damping $\gamma_{\rm b}$ of the mechanical motion is much smaller than its own natural frequency $\omega_{\rm m}$. If $\gamma_{\rm b} \ll \omega_{\rm m}$, the set of frequencies contributing to $\hat{b}[\Omega_{\rm k}]$ and $\hat{b}^{\dagger}[\Omega_{\rm k}]$ will be located around $\omega_{\rm m}$ and $-\omega_{\rm m}$ respectively. This, in turn, implies that we can substitute the effective damping $\Gamma_{\rm b}(\Omega_{\rm k})$ and shift $\omega_{\rm b}(\Omega_{\rm k})$ with the values taken at the relevant frequencies $\pm\omega_{\rm m}$. Defining the constant effective decay $\Gamma_{\rm b} = \Gamma_{\rm b}(\omega_{\rm m}) = \Gamma_{\rm b}(-\omega_{\rm m})$ and frequency shift $\omega_{\rm b} = \omega_{\rm b}(\omega_{\rm m}) = -\omega_{\rm b}(-\omega_{\rm m})$, we can rewrite Supplementary Eq. (72) in the following way:

$$
\begin{aligned}
\hat{b}\left[\Omega_{\rm k}\right]\left(\frac{\gamma_{\rm b}+\Gamma_{\rm b}}{2} - i\left[\Omega_{\rm k}-\omega_{\rm m}-\omega_{\rm b}\right]\right) = i\frac{x_0}{\hbar}\hat{F}_{\rm b}\left[\Omega_{\rm k}\right] + \frac{2g_1\alpha}{\gamma_{\rm t}+\gamma_{\rm r}}\omega_{\rm a}^2\sqrt{\frac{\gamma_{\rm t}C_0}{\hbar T\omega_{\rm s}}}\times \\
\times\left(\frac{\hat{V}_{\rm R2}\left[\Omega_{\rm k}-\omega_{\rm s}\right]-\hat{V}_{\rm R1}\left[\Omega_{\rm k}-\omega_{\rm s}\right]}{-\omega_{\rm a}^2+\left(\Omega_{\rm k}-\omega_{\rm s}\right)\left(i\gamma_l+\Omega_{\rm k}-\omega_{\rm s}\right)} - \frac{\hat{V}_{\rm R2}\left[\Omega_{\rm k}+\omega_{\rm s}\right]-\hat{V}_{\rm R1}\left[\Omega_{\rm k}+\omega_{\rm s}\right]}{-\omega_{\rm a}^2+\left(\Omega_{\rm k}+\omega_{\rm s}\right)\left(i\gamma_l+\Omega_{\rm k}+\omega_{\rm s}\right)}\right),
\end{aligned}
\tag{75}
$$

from which we can see that $\Gamma_{\rm b}$ affects the dynamics as an effective decay and $\omega_{\rm b}$ serves as a frequency shift.

We can now take the Fourier series of Supplementary Eq. (75), in order to go back to the time domain, and solve formally for $\hat{b}(t)$. Defining the noise operator $\hat{N}(t) = \hat{N}^{\dagger}(t)$ to be

$$
\hat{N}(t) = \sum_{k=-\infty}^{\infty}\hat{N}\left[\Omega_{\rm k}\right]\frac{e^{-i\Omega_{\rm k}t}}{\sqrt{T}},
\tag{76}
$$

$$\hat{N}\left[\Omega_{\mathrm{k}}\right]=\left(\frac{\hat{V}_{\mathrm{R}2}\left[\Omega_{\mathrm{k}}-\omega_{\mathrm{s}}\right]-\hat{V}_{\mathrm{R}1}\left[\Omega_{\mathrm{k}}-\omega_{\mathrm{s}}\right]}{-\omega_{\mathrm{a}}^{2}+\left(\Omega_{\mathrm{k}}-\omega_{\mathrm{s}}\right)\left(i\gamma_{l}+\Omega_{\mathrm{k}}-\omega_{\mathrm{s}}\right)}-\frac{\hat{V}_{\mathrm{R}2}\left[\Omega_{\mathrm{k}}+\omega_{\mathrm{s}}\right]-\hat{V}_{\mathrm{R}1}\left[\Omega_{\mathrm{k}}+\omega_{\mathrm{s}}\right]}{-\omega_{\mathrm{a}}^{2}+\left(\Omega_{\mathrm{k}}+\omega_{\mathrm{s}}\right)\left(i\gamma_{l}+\Omega_{\mathrm{k}}+\omega_{\mathrm{s}}\right)}\right), \tag{77}$$

we obtain

$$\dot{\hat{b}}(t)=\hat{b}(t)\left(-i\left(\omega_{\mathrm{m}}+\omega_{\mathrm{b}}\right)-\frac{\gamma_{\mathrm{b}}+\Gamma_{\mathrm{b}}}{2}\right)+i\frac{x_{0}}{\hbar}\hat{F}_{\mathrm{b}}(t)+\frac{2g_{1}\alpha}{\gamma_{\mathrm{t}}+\gamma_{\mathrm{r}}}\omega_{\mathrm{a}}^{2}\sqrt{\frac{\gamma_{\mathrm{t}}C_{0}}{T\omega_{\mathrm{s}}\hbar}}\hat{N}(t). \tag{78}$$

From Supplementary Eq. (78) it is now possible to obtain the exact solution for $\hat{b}(t)$ as a function of the quantum noises $\hat{F}_{\mathrm{b}}(t)$ and $\hat{N}(t)$:

$$\begin{aligned}\hat{b}(t)=\hat{b}(0)e^{-\left[\frac{\gamma_{\mathrm{b}}+\Gamma_{\mathrm{b}}}{2}+i(\omega_{\mathrm{m}}+\omega_{\mathrm{b}})\right]t}&+i\frac{x_{0}}{\hbar}\int_{0}^{\mathrm{t}}e^{-\left[\frac{\gamma_{\mathrm{b}}+\Gamma_{\mathrm{b}}}{2}+i(\omega_{\mathrm{m}}+\omega_{\mathrm{b}})\right](t-\tau_{1})}\hat{F}_{\mathrm{b}}(\tau_{1})d\tau_{1}\\&+\frac{2g_{1}\alpha}{\gamma_{\mathrm{t}}+\gamma_{\mathrm{r}}}\omega_{\mathrm{a}}^{2}\sqrt{\frac{\gamma_{\mathrm{t}}C_{0}}{T\omega_{\mathrm{s}}\hbar}}\int_{0}^{\mathrm{t}}e^{-\left[\frac{\gamma_{\mathrm{b}}+\Gamma_{\mathrm{b}}}{2}+i(\omega_{\mathrm{m}}+\omega_{\mathrm{b}})\right](t-\tau_{1})}\hat{N}(\tau_{1})d\tau_{1},\end{aligned} \tag{79}$$

where $\hat{b}(0)$ is the annihilation operator at the initial time $t=0$. From here one can verify that the mechanical creation and annihilation operators satisfy the standard commutation relation, $\left[\hat{b}(t),\hat{b}^{\dagger}(t)\right]=1$.

With the above results, we can finally determine $n_{\mathrm{b}}(t)$ to be

$$\begin{aligned}n_{\mathrm{b}}(t)&=n_{\mathrm{b}}(0)e^{-(\gamma_{\mathrm{t}}+\gamma_{\mathrm{r}})t}\\&+\frac{1}{2\hbar m\omega_{\mathrm{m}}}\int_{0}^{\mathrm{t}}\int_{0}^{\mathrm{t}}e^{-\frac{\gamma_{\mathrm{b}}+\Gamma_{\mathrm{b}}}{2}(2t-\tau_{1}-\tau_{2})}e^{-i(\omega_{\mathrm{m}}+\omega_{\mathrm{b}})(\tau_{1}-\tau_{2})}\left\langle\hat{F}_{\mathrm{b}}^{\dagger}(\tau_{1})\hat{F}_{\mathrm{b}}(\tau_{2})\right\rangle d\tau_{1}d\tau_{2}\\&+4\frac{g_{1}^{2}|\alpha|^{2}\omega_{\mathrm{a}}^{4}}{\left(\gamma_{\mathrm{t}}+\gamma_{\mathrm{r}}\right)^{2}}\frac{\gamma_{\mathrm{r}}C_{0}}{T\omega_{\mathrm{s}}\hbar}\int_{0}^{\mathrm{t}}\int_{0}^{\mathrm{t}}e^{-\frac{\gamma_{\mathrm{b}}+\Gamma_{\mathrm{b}}}{2}(2t-\tau_{1}-\tau_{2})}e^{-i(\omega_{\mathrm{m}}+\omega_{\mathrm{b}})(\tau_{1}-\tau_{2})}\left\langle\hat{N}(\tau_{1})\hat{N}(\tau_{2})\right\rangle d\tau_{1}d\tau_{2}.\end{aligned} \tag{80}$$

At this stage, the whole problem is reduced to computing the variances of the mechanical noise $\left\langle\hat{F}_{\mathrm{b}}^{\dagger}(\tau_{1})\hat{F}_{\mathrm{b}}(\tau_{2})\right\rangle$ and the electric fields $\left\langle\hat{N}(\tau_{1})\hat{N}(\tau_{2})\right\rangle$. As a consequence of our assumption $\gamma_{\mathrm{b}}\ll\omega_{\mathrm{m}}$, we can neglect off–resonant contributions in $\left\langle\hat{F}_{\mathrm{b}}^{\dagger}(\tau_{1})\hat{F}_{\mathrm{b}}(\tau_{2})\right\rangle$ and write it as

$$\left\langle\hat{F}_{\mathrm{b}}^{\dagger}(\tau_{1})\hat{F}_{\mathrm{b}}(\tau_{2})\right\rangle\simeq2\hbar m\omega_{\mathrm{m}}\gamma_{\mathrm{b}}\bar{n}_{\mathrm{m}}\delta(\tau_{2}-\tau_{1}). \tag{81}$$

The determination of the other variance, $\left\langle\hat{N}(\tau_{1})\hat{N}(\tau_{2})\right\rangle$, is more involved. In general, taking the definition of $\hat{N}(t)$ in Supplementary Eqs. (76), (77) and substituting it into expression for the correlator, one encounters a number of products of the form

$$\frac{\left\langle\hat{V}_{\mathrm{Ri}}\left[\Omega_{h}\right]\hat{V}_{Rj}\left[\Omega_{\mathrm{m}}\right]\right\rangle e^{-i\Omega_{h}t}e^{-i\Omega_{\mathrm{m}}t}}{\mathrm{Den}\left(\Omega_{h},\Omega_{\mathrm{m}}\right)}, \tag{82}$$

where $i,j=1,2$, and Ω_{h}, Ω_{m} are generic Fourier variables. The denominators $\mathrm{Den}\left(\Omega_{h},\Omega_{\mathrm{m}}\right)$ can be determined from Supplementary Eq. (77). Considering that

$$\left\langle\hat{V}_{\mathrm{Ri}}\left[\Omega_{h}\right]\hat{V}_{Rj}\left[\Omega_{\mathrm{m}}\right]\right\rangle=0,\quad\forall i\neq j, \tag{83a}$$

$$\begin{aligned}\left\langle\hat{V}_{\mathrm{Ri}}\left[\Omega_{h}\right]\hat{V}_{\mathrm{Ri}}\left[\Omega_{\mathrm{m}}\right]\right\rangle=&\frac{\hbar\Omega_{h}R}{2}\bar{n}_{\mathrm{e}}(\Omega_{h},T_{\mathrm{e}})\delta\left(\Omega_{h}+\Omega_{\mathrm{m}}\right)\theta\left(-\Omega_{h}\right)\\&+\frac{\hbar\Omega_{h}R}{2}\left[\bar{n}_{\mathrm{e}}(\Omega_{h},T_{\mathrm{e}})+1\right]\delta\left(\Omega_{h}+\Omega_{\mathrm{m}}\right)\theta\left(\Omega_{h}\right),\end{aligned} \tag{83b}$$

with $\bar{n}_{\mathrm{e}}(\Omega_{h},T_{\mathrm{e}})$ being the occupation number of the electrical reservoir at the considered frequency Ω_{h} and temperature T_{e}, we get

$$\begin{aligned}\left\langle\hat{N}(\tau_{1})\hat{N}(\tau_{2})\right\rangle=&\frac{(\omega_{\mathrm{s}}-\omega_{\mathrm{m}})\hbar R\bar{n}_{\mathrm{e}}\left(\omega_{\mathrm{s}}-\omega_{\mathrm{m}},T_{\mathrm{e}}\right)\delta\left(\tau_{2}-\tau_{1}\right)}{\omega_{\mathrm{a}}^{4}-2\omega_{\mathrm{a}}^{2}\left(\omega_{\mathrm{m}}-\omega_{\mathrm{s}}\right)^{2}+\left(\omega_{\mathrm{m}}-\omega_{\mathrm{s}}\right)^{2}\left(\gamma_{l}^{2}+\left(\omega_{\mathrm{m}}-\omega_{\mathrm{s}}\right)^{2}\right)}\\&+\frac{(\omega_{\mathrm{s}}+\omega_{\mathrm{m}})\hbar R\bar{n}_{\mathrm{e}}\left(\omega_{\mathrm{s}}+\omega_{\mathrm{m}},T_{\mathrm{e}}\right)\delta\left(\tau_{2}-\tau_{1}\right)}{\omega_{\mathrm{a}}^{4}-2\omega_{\mathrm{a}}^{2}\left(\omega_{\mathrm{m}}+\omega_{\mathrm{s}}\right)^{2}+\left(\omega_{\mathrm{m}}+\omega_{\mathrm{s}}\right)^{2}\left(\gamma_{l}^{2}+\left(\omega_{\mathrm{m}}+\omega_{\mathrm{s}}\right)^{2}\right)}.\end{aligned} \tag{84}$$

We have here assumed both resistances R to be at the same temperature T_{e}, and neglected fast–oscillating contributions to $\left\langle \hat{N}(\tau_1)\hat{N}(\tau_2)\right\rangle$. In Supplementary Eq. (83b), we also denoted the Heaviside step function with the letter θ.

With this last result, we are finally able to determine the time evolution for the average phonon number operator $n_{\mathrm{b}}(t)$. In fact, using equations (81) and (84), it is possible to calculate the integrals in Supplementary Eq. (80) and therefore obtain a clear analytical expression for $n_{\mathrm{b}}(t)$. In its most general form, the expression is rather involved. For simplicity, we restrict ourselves to a reasonable approximation that allows us to better understand the final result. Considering the limit $\omega_{\mathrm{a}} \gg \omega_{\mathrm{s}} \gg \omega_{\mathrm{m}}$, from Supplementary Eqs. (73) and (74) with $\Omega_{\mathrm{k}} \to \omega_{\mathrm{m}}$, we can rewrite the effective decay Γ_{b} and the frequency shift ω_{b} to be

$$\Gamma_{\mathrm{b}} = \frac{8g_1^2|\alpha|^2\gamma_l\gamma_{\mathrm{t}}\omega_{\mathrm{m}}}{T\left(\gamma_{\mathrm{t}}+\gamma_{\mathrm{r}}\right)^2\omega_{\mathrm{s}}\omega_{\mathrm{a}}^2}, \tag{85}$$

$$\omega_{\mathrm{b}} = -\frac{4g_1^2|\alpha|^2\gamma_{\mathrm{t}}}{T\left(\gamma_{\mathrm{t}}+\gamma_{\mathrm{r}}\right)^2\omega_{\mathrm{s}}}. \tag{86}$$

From now on, referring to Γ_{b} and ω_{b}, we implicitly consider the form given in these last two equations. Note, however, that Supplementary Eq. (73) describes the damping due to the difference in sideband strengths for a mode at frequency ω_{a} driven at frequency ω_{s}. In the limit where ω_{a} is large, this induced damping is very small. The shift of Supplementary Eq. (74) may be sizeable, but merely leads to a new mechanical resonance frequency, which is not important for the present discussion. Furthermore, as a consequence of $\omega_{\mathrm{s}} \gg \omega_{\mathrm{m}}$, we assume that $\bar{n}_{\mathrm{e}}(\omega_{\mathrm{s}}-\omega_{\mathrm{m}},T_{\mathrm{e}})$ and $\bar{n}_{\mathrm{e}}(\omega_{\mathrm{s}}+\omega_{\mathrm{m}},T_{\mathrm{e}})$ are approximatively equal in Supplementary Eq. (84), such that

$$\bar{n}_{\mathrm{e}}(\omega_{\mathrm{s}}\pm\omega_{\mathrm{m}},T_{\mathrm{e}}) \simeq \bar{n}_{\mathrm{e}}(\omega_{\mathrm{s}},T_{\mathrm{e}}) \equiv \bar{n}_{\mathrm{e}}. \tag{87}$$

Putting all these results together we can finally derive $n_{\mathrm{b}}(t)$ to be

$$n_{\mathrm{b}}(t) = n_{\mathrm{b}}(0)e^{-(\gamma_{\mathrm{b}}+\Gamma_{\mathrm{b}})t} + \frac{\gamma_{\mathrm{b}}}{\gamma_{\mathrm{b}}+\Gamma_{\mathrm{b}}}\bar{n}_{\mathrm{m}}\left(1-e^{-(\gamma_{\mathrm{b}}+\Gamma_{\mathrm{b}})t}\right) + \frac{\Gamma_{\mathrm{b}}}{\gamma_{\mathrm{b}}+\Gamma_{\mathrm{b}}}\frac{\omega_{\mathrm{s}}}{\omega_{\mathrm{m}}}\left(\bar{n}_{\mathrm{e}}+\frac{1}{2}\right)\left(1-e^{-(\gamma_{\mathrm{b}}+\Gamma_{\mathrm{b}})t}\right). \tag{88}$$

As a conclusive part of this discussion, let us determine Δn_{b}. Recalling that Δn_{b} is the change in the phonon number for the membrane initially cooled in its ground state, we get

$$\Delta n_{\mathrm{b}} = \gamma_{\mathrm{b}}\bar{n}_{\mathrm{m}}T + \frac{\omega_{\mathrm{s}}}{\omega_{\mathrm{m}}}\Gamma_{\mathrm{b}}\left(\bar{n}_{\mathrm{e}}+\frac{1}{2}\right)T, \tag{89}$$

where we truncated the expansion at first order in T. This is the parameter used as denominator in $\lambda_{\mathrm{b}} = D^2/\Delta n_{\mathrm{b}}$, presented in the main text neglecting the contribution from the mechanical reservoir.

The parameter Δn_{b} derived above takes into account only the heating induced by the redistribution of charges on the capacitor. Asymmetries are well described analytically by considering a linear coupling δg_1 and capacitance δC such that $C^{-1}(\pm\hat{x}) \propto (C_0 \pm \delta C)^{-1} \pm (g_1 \pm \delta g_1)\hat{x} + g_2\hat{n}_{\mathrm{b}}$. These are the only asymmetries directly affecting the electromechanical coupling; other ones only enter as higher order perturbation, as we will see later on. In this case we can find an overall residual linear coupling $g_{\mathrm{r}} = \frac{1}{2}C_0x_0\omega_{\mathrm{s}}\partial_x\left[C(x)+C(-x)\right]^{-1} > 0$ of the membrane equal to

$$g_{\mathrm{r}} = \delta g_1 + 2\frac{g_1\delta C}{C_0} + \frac{\delta g_1\delta C^2}{C_0^2}. \tag{90}$$

Under the assumption that the parasitic elements R and L of the circuit are smaller than the non-parasitic ones R_0 and L_0, we can neglect the coupling to the antisymmetric mode to lowest order (this mode is far off resonant, while the symmetric one is resonant). The heating is then dominated by the residual linear coupling g_{r}, similarly to the situation encountered above in the Supplementary note 1. We can thus use the result in Supplementary Eq. (40) obtained for the RLC circuit to identify the contribution to the heating:

$$n_{\mathrm{b}}(t) = \frac{\tilde{\Gamma}_{\mathrm{b}}}{\gamma_{\mathrm{b}}+\Gamma_{\mathrm{b}}}(1+2\bar{n}_{\mathrm{e}})\left(1-e^{-(\gamma_{\mathrm{b}}+\Gamma_{\mathrm{b}})t}\right). \tag{91}$$

Here, $\tilde{\Gamma}_{\mathrm{b}}$ has been defined from Γ_{b} in Supplementary Eq. (41) by substituting g_1 with g_{r}:

$$\tilde{\Gamma}_{\mathrm{b}} = \frac{4g_{\mathrm{r}}^2|\alpha|^2\gamma_{\mathrm{t}}}{T(\gamma_{\mathrm{t}}+\gamma_{\mathrm{r}})\left[(\gamma_{\mathrm{t}}+\gamma_{\mathrm{r}})^2+4\omega_{\mathrm{m}}^2\right]}. \tag{92}$$

From Supplementary Eq. (91) we can determine $\Delta n_{\rm b}$,

$$\Delta n_{\rm b} = \tilde{\Gamma}_{\rm b}(1+2\bar{n}_{\rm e})T, \tag{93}$$

being the denominator of the parameter $\lambda_{\rm p} = D^2/\Delta n_{\rm b}$ presented in the main text for $\delta C \to 0$.

Putting together Supplementary Eqs. (88) and (91), we can find the time evolution of the average phonon number $n_{\rm b}(t)$, taking into account all sources of heating:

$$n_{\rm b}(t) = n_{\rm b}(0)e^{-(\gamma_{\rm b}+\Gamma_{\rm b})t} + \left[\gamma_{\rm b}\bar{n}_{\rm m} + \left(\Gamma_{\rm b}\frac{\omega_{\rm s}}{2\omega_{\rm m}} + \tilde{\Gamma}_{\rm b}\right)(1+2\bar{n}_{\rm e})\right]\frac{1-e^{-(\gamma_{\rm b}+\Gamma_{\rm b})t}}{\gamma_{\rm b}+\Gamma_{\rm b}}. \tag{94}$$

This result will be supported by our simulations, presented in the following.

Heating simulation

In this subsection we present two methods for simulating the membrane heating, described by $n_{\rm b}(t)$. For the double arm circuit in supplementary Fig. 4 we cannot employ the wave function Monte–Carlo approach [8] that was used for supplementary Fig. 3. Quantum jump simulations apply to Markovian reservoirs, and thereby assume that there is the same number of thermal photons for all frequencies. In our case, there is a big difference in the resonance frequencies of the symmetric $\omega_{\rm s}$ and antisymmetric $\omega_{\rm a}$ electrical modes. Therefore, we need to address the frequency dependence of the reservoir occupation. At a given temperature $T_{\rm e}$ of the electrical reservoirs, the antisymmetric mode will come to equilibrium with $\bar{n}_{\rm e}(\omega_{\rm a}, T_{\rm e}) = [\exp(\hbar\omega_{\rm a}/k_B T_{\rm e}) - 1]^{-1}$ photons at frequency $\omega_{\rm a}$. On the other hand, the coupling between the mechanical oscillator and the antisymmetric field is mediated by the driving, which is resonant with the symmetric mode. Hence, the membrane couples to a reservoir containing $\bar{n}_{\rm e}(\omega_{\rm s}, T_{\rm e}) \gg \bar{n}_{\rm e}(\omega_{\rm a}, T_{\rm e})$ photons, as indicated in Supplementary Eq. (88). Therefore, more advanced methods are required for simulating the present situation.

We will investigate the approximations taken for deriving Supplementary Eq. (94) on two different levels. First, we consider Supplementary Eqs. (69), (70), (71) and solve them in the Fourier domain, keeping the off-resonant terms that have been ignored in the derivation of Supplementary Eq. (88) above. Second, we allow for any possible deviation from the balanced circuit in supplementary Fig. 4, by introducing unbalanced parasitic resistances R_1 and R_2, inductances L_1 and L_2, rest capacitances C_1 and C_2, and linear couplings $g_1 + \delta g_1$ and $g_1 - \delta g_1$. In the following, since we are interested in the time evolution of the mechanical subsystem for times longer than T, we will explicitly consider a time variable $\tau > T$ in the Fourier expansion of Supplementary Eq. (9)

Let us start our analysis by rewriting Supplementary Eqs. (69), (70), and (71) with the creation $\hat{a}^\dagger$ and annihilation $\hat{a}$ operators for the electrical charge $\hat{Q}_{\rm a}$ and flux $\hat{\Phi}_{\rm a}$:

$$\begin{aligned}\hat{a}[\Omega_{\rm k}]\left(-i\Omega_{\rm k} - i\omega_{\rm a} + \frac{\gamma_l}{2}\right) =& \frac{\gamma_l}{2}\hat{a}^\dagger[\Omega_{\rm k}] - i\frac{g_1\alpha}{\gamma_{\rm r}+\gamma_{\rm t}}\sqrt{\frac{\gamma_{\rm t}\omega_{\rm a}}{T\omega_{\rm s}}}\left(\hat{b}[\Omega_{\rm k}-\omega_{\rm s}] + \hat{b}^\dagger[\Omega_{\rm k}-\omega_{\rm s}] - \hat{b}[\Omega_{\rm k}+\omega_{\rm s}] - \hat{b}^\dagger[\Omega_{\rm k}+\omega_{\rm s}]\right)\\ &+ i\sqrt{\frac{C_0\omega_{\rm a}}{\hbar}}\left(\hat{V}_{\rm R2}[\Omega_{\rm k}] - \hat{V}_{\rm R1}[\Omega_{\rm k}]\right) + \frac{\hat{a}(t=0)}{\sqrt{\tau}} - \frac{\hat{a}(t=\tau)}{\sqrt{\tau}},\end{aligned} \tag{95}$$

$$\begin{aligned}\hat{b}[\Omega_{\rm k}]\left(-i\Omega_{\rm k} - i\omega_{\rm m} + \frac{\gamma_{\rm b}}{2}\right) =& -i\frac{g_1\alpha}{\gamma_{\rm r}+\gamma_{\rm t}}\sqrt{\frac{\gamma_{\rm t}\omega_{\rm a}}{T\omega_{\rm s}}}\left(\hat{a}[\Omega_{\rm k}-\omega_{\rm s}] + \hat{a}^\dagger[\Omega_{\rm k}-\omega_{\rm s}] - \hat{a}[\Omega_{\rm k}+\omega_{\rm s}] - \hat{a}^\dagger[\Omega_{\rm k}+\omega_{\rm s}]\right)\\ &+ i\frac{x_0}{\hbar}\hat{F}_{\rm b}[\Omega_{\rm k}] + \frac{\hat{b}(t=0)}{\sqrt{\tau}} - \frac{\hat{b}(t=\tau)}{\sqrt{\tau}},\end{aligned} \tag{96}$$

where $\hat{Q}_{\rm a} = \sqrt{\hbar C_0\omega_{\rm a}}(\hat{a}+\hat{a}^\dagger)/2$ and $\hat{\Phi}_{\rm a} = i\sqrt{\hbar/(C_0\omega_{\rm a})}(\hat{a}^\dagger - \hat{a})$. Note that in Supplementary Eqs. (95) and (96) we have ignored g_2 contributions ($g_2 \ll g_1$), and added boundary terms, that are required to describe non–periodic dynamics in the Fourier series. Without those, we would obtain the steady state evolution, that cannot capture the thermalisation from the ground or any other state than the equilibrium. The additional terms can be adjusted to represent the desired initial state.

Above, we truncated the Fourier series by keeping only the terms resonant with the mechanical frequency $\omega_{\rm m}$. Instead, we will now include additional terms at frequencies centred around $\pm\omega_{\rm m}$,

$$\hat{O}(t) = \sum_{k=-N_{\rm j}}^{N_{\rm j}}\sum_{l=-N_{\rm f}}^{N_{\rm f}}\hat{O}\left[\omega_{\rm m} + k\omega_{\rm s} + l\frac{2\pi}{\tau}\right]\frac{e^{-i\left(\omega_{\rm m}+k\omega_{\rm s}+l\frac{2\pi}{\tau}\right)}}{\sqrt{\tau}} + \hat{O}\left[-\omega_{\rm m} - k\omega_{\rm s} - l\frac{2\pi}{\tau}\right]\frac{e^{i\left(\omega_{\rm m}+k\omega_{\rm s}+l\frac{2\pi}{\tau}\right)}}{\sqrt{\tau}}, \tag{97}$$

where $\hat{O}$ can be any operator $\hat{a}^{(\dagger)}$ or $\hat{b}^{(\dagger)}$, and k, l are integers corresponding to the expansion. In Supplementary Eq. (97), the parameter N_{j} indicates how many sidebands we consider, while N_{f} is related with the convergence of the Fourier series. By setting $N_{\mathrm{j}} = 0$, we are assuming that the mechanical and electrical subsystems are completely decoupled, while $N_{\mathrm{j}} = 2$ is the case studied above analytically. Corrections are given for $N_{\mathrm{j}} > 2$. An example of the considered frequencies is illustrated in supplementary Fig. 5 for $N_{\mathrm{j}} = 2$ and $N_{\mathrm{f}} = 4$. The Fourier components

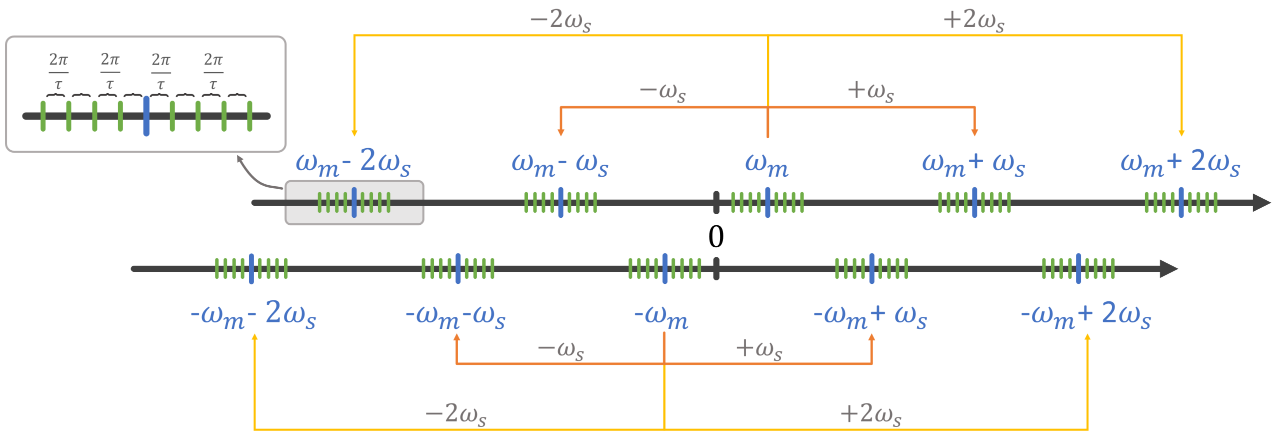

Supplementary Fig. 5: **Relevant frequencies for the heating simulation**. Example for a possible set of relevant frequencies in the truncated Fourier series of Supplementary Eq. (97). In this particular case, we choose $N_{\mathrm{j}} = 2$ and $N_{\mathrm{f}} = 4$. In the upper arrow the frequencies obtained starting from ω_{m}, in the lower arrow from $-\omega_{\mathrm{m}}$.

$\hat{a}\left[\pm\left(\omega_{\mathrm{m}} + k\omega_{\mathrm{s}} + l\frac{2\pi}{\tau}\right)\right]$ and $\hat{b}\left[\pm\left(\omega_{\mathrm{m}} + k\omega_{\mathrm{s}} + l\frac{2\pi}{\tau}\right)\right]$ are then derived from the set of coupled linear equations (95) and (96), that can be solved efficiently using the matrix notation. A comparison between the result of Supplementary Eq. (88) and the so–derived simulations is presented in supplementary Fig. 6. In the left plot we present $T_{1/2}$ for different values of the linear coupling g_1. Here, $T_{1/2}$ is the time for which the average mechanical occupation is half the one of the steady state $n_{\mathrm{b}}(t \to \infty)$. In the right plot, it is possible to see the g_1–dependence of the mechanical steady state population. As it is possible to see, the simulations converge to the analytical curve of Supplementary Eq. (88) independently from the parameter N_{j}. This means that off-resonant terms do not play a major role, and that the approximations taken for deriving Supplementary Eq. (88) are appropriate. The small discrepancy between analytical and simulated results is dominated by a poor convergence of the Fourier series. By taking bigger N_{f}, this discrepancy would be eventually removed.

In the second part of this subsection we simulate the electromechanical system presented in supplementary Fig. 4, allowing all the parasitic elements to differ between the two arms. In particular, we introduce deviations for resistances $R \to R \pm \delta R$, inductances $L \to L \pm \delta L$, bare capacitances $C_0 \to C_0 \pm \delta C$, and the linear couplings $g_1 \to g_1 \pm \delta g_1$ in the left and right arms. Once we have done this, following the standard approach presented above we can derive the equations of motion of our electromechanical setup to be:

$$\dot{\hat{Q}}_{\mathrm{a}} = \frac{\hat{\Phi}_{\mathrm{a}}}{2L} + \frac{\delta L^2 \hat{\Phi}_{\mathrm{a}} - 2L\delta L \hat{\Phi}_{\mathrm{s}}}{2L\left(L^2 + 2LL_0 - \delta L^2\right)}, \tag{98}$$

$$\begin{aligned}\dot{\hat{\Phi}}_{\mathrm{a}} = &-\frac{2\hat{Q}_{\mathrm{a}}}{C_0} - \frac{g_1}{C_0\omega_{\mathrm{s}}}\hat{Q}_{\mathrm{s}}\left(\hat{b} + \hat{b}^\dagger\right) - \frac{2g_2}{C_0\omega_{\mathrm{s}}}\hat{Q}_{\mathrm{a}}\hat{b}^\dagger\hat{b} - \gamma_l\hat{\Phi}_{\mathrm{a}} + 2\left(\hat{V}_{\mathrm{R2}} - \hat{V}_{\mathrm{R1}}\right) - \frac{2\delta C^2\hat{Q}_{\mathrm{a}}}{C_0\left(C_0^2 - \delta C^2\right)} + \frac{\delta C\hat{Q}_{\mathrm{s}}}{C_0^2 - \delta C^2} \\ &- \frac{2\delta g_1}{C_0\omega_{\mathrm{s}}}\hat{Q}_{\mathrm{a}}\left(\hat{b} + \hat{b}^\dagger\right) + \frac{\delta L(-R\delta L + L\delta R)}{L\left(L^2 + 2LL_0 - \delta L^2\right)}\hat{\Phi}_{\mathrm{a}} + \frac{2R\delta L - 2L\delta R}{L^2 + 2LL_0 - \delta L^2}\hat{\Phi}_{\mathrm{s}},\end{aligned} \tag{99}$$

$$\dot{\hat{Q}}_{\mathrm{s}} = \frac{2\hat{\Phi}_{\mathrm{s}}}{L + 2L_0} - \frac{\delta L\hat{\Phi}_{\mathrm{a}}}{\left(L^2 + 2LL_0 - 2\delta L^2\right)} + \frac{2\delta L^2\hat{\Phi}_{\mathrm{s}}}{\left(L + 2L_0\right)\left(L^2 + 2LL_0 - 2\delta L^2\right)}, \tag{100}$$

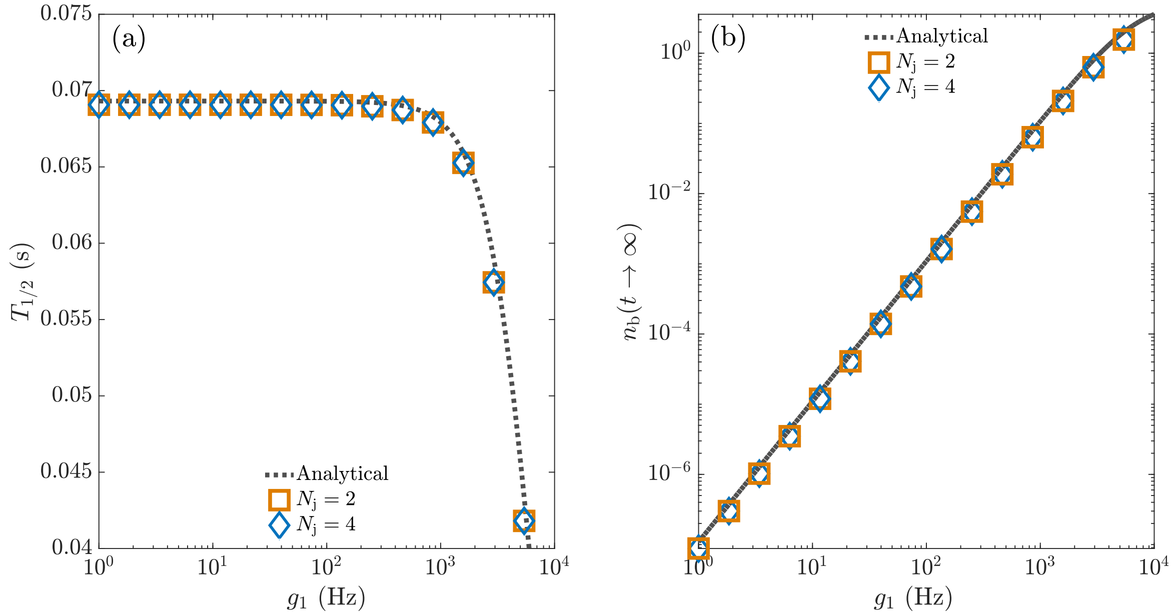

Supplementary Fig. 6: **Comparison between Supplementary Eq.** (88) **and simulations**. In **(a)**, we show $T_{1/2}$ versus the linear coupling g_1, with $T_{1/2}$ defined by the relation $n_b(T_{1/2}) = n_b(t \to \infty)/2$. In **(b)**, the average mechanical steady state population $n_b(t \to \infty)$ is presented, as a function of g_1. Dotted lines are the analytical predictions obtained from Supplementary Eq. (88). Red squares and blue diamonds come from simulations. The first one corresponds to the case where we allow for only one sideband ($N_j = 2$), and the second for two ($N_j = 4$). As it is possible to see, two–sidebands corrections are negligible. We assumed $L/L_0 = 10^{-4}$, $R/Z_{out} = 0.1$, $\omega_s = (2\pi)3$ GHz, $\omega_m = (2\pi)314$ MHz, $\gamma_t \simeq \gamma_r = (2\pi)100$ kHz, $\gamma_b = (2\pi)10$ Hz, $|\alpha|^2/\tau = 10^{12}$ s^{-1} and $\bar{n}_e = \bar{n}_m = 0$. Here, τ is the arbitrary time of the Fourier series in Supplementary Eq. (9).

$$\begin{aligned}\dot{\hat{\Phi}}_s =& -\frac{\hat{Q}_s}{2C_0} - \frac{g_1}{C_0\omega_s}\hat{Q}_a\left(\hat{b}+\hat{b}^\dagger\right) - \frac{2g_2}{C_0\omega_s}\hat{Q}_s\hat{b}^\dagger\hat{b} - (\gamma_r+\gamma_t)\hat{\Phi}_s + 2\left(\hat{V}_{in}+\hat{V}_{R0}\right) + \hat{V}_{R1} + \hat{V}_{R2} \\ &+ \frac{\delta C\hat{Q}_a}{C_0^2-\delta C^2} - \frac{\delta C^2\hat{Q}_s}{2C_0\left(C_0^2-\delta C^2\right)} - \frac{\delta g_1}{2C_0\omega_s}\hat{Q}_s\left(\hat{b}+\hat{b}^\dagger\right) + \frac{R\delta L + 2(R_0+Z_{out})\delta L - (L+2L_0)\delta R}{2\left(L^2+2LL_0-\delta L^2\right)}\hat{\Phi}_a \\ &+ \frac{\delta L\left[\delta R(L+2L_0) - (R+2(R_0+Z_{out}))\,\delta L\right]}{(L+2L_0)\left(L^2+2LL_0-\delta L^2\right)}\hat{\Phi}_s,\end{aligned} \tag{101}$$

$$\dot{\hat{b}} = -i\omega_m\hat{b} - i\frac{g_1}{\hbar C_0\omega_s}\hat{Q}_a\hat{Q}_s - i\frac{g_2}{\hbar C_0\omega_s}\left(\hat{b}+\hat{b}^\dagger\right)\left(\hat{Q}_a^2+\frac{\hat{Q}_s^2}{4}\right) - \frac{\gamma_b}{2}\hat{b} + i\frac{x_0}{\hbar}\hat{F}_b + \frac{i\delta g_1}{\hbar C_0\omega_s}\left(\hat{Q}_a^2+\frac{\hat{Q}_s^2}{4}\right). \tag{102}$$

For simulating this set of equations we follow a similar approach to the one presented above, where we neglected off–resonant terms that do not contribute substantially to the mechanical heating and the quadratic coupling (since $g_2 \ll g_1$). In order to get a solution, we have linearised the symmetric and antisymmetric electrical fields, and the mechanical operators. As opposed to the previous investigations, now our simulations start at a specific time. We thus consider transient dynamics for the average mechanical operators, that we can set to have any initial value (generally zero, as for the ground state). Our simulations are thus performed with the mechanical average values being time dependent [3]. As it is possible to see from supplementary Fig. 7, the agreement between the analytical results and the simulations is excellent, as far as the asymmetries δR, δL and δC are small enough. The system is more susceptible to relative increase of δC as compared to δR and δL. This is because δR and δL are asymmetries of parasitic elements of the circuit, while δC affects the main (and only) capacitor. A non–vanishing value of δC directly affects the residual linear coupling g_r [see Supplementary Eq. (90)] and thus has a larger influence, that is included in our analytical prediction of Supplementary Eq. (94). Roughly speaking, whenever $\delta C/C$, $\delta R/R$ and $\delta L/L$ are smaller than 25%, the agreement between Supplementary Eq. (94) and the simulations is very good.

In summary, we have tested the assumptions made in the derivation of our analytical results. We have proven with the Fourier analysis that off–resonant terms do not contribute significantly to the mechanical heating, while

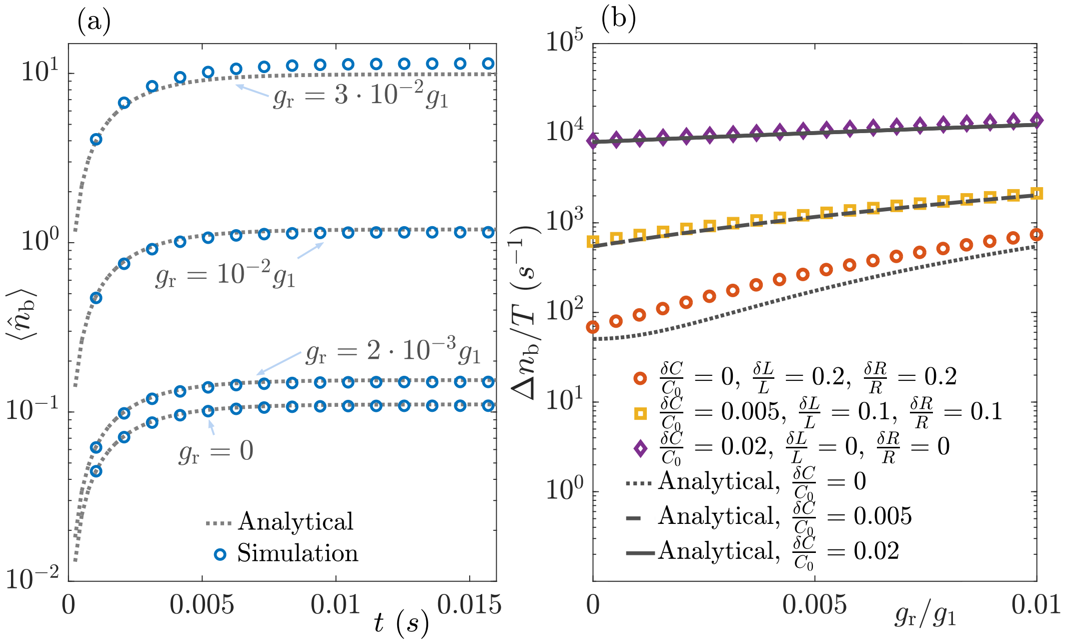

Supplementary Fig. 7: **Heating in the unbalanced case**. **(a)** Comparison between the analytical curves given by Supplementary Eq. (94) and the full simulations of the system (98), (99), (100), (101), and (102). From the bottom to the top we have set $\delta g_1/g_1$ to be 0, $2 \cdot 10^{-3}$, 10^{-2} and $3 \cdot 10^{-2}$. We use $\delta R = \delta L = \delta C = 0$. **(b)** Heating rate $\Delta n_b/T = \partial_t n_b(t)$ as a function of the normalised residual linear coupling $\delta g_1/g_1$. Here we test Supplementary Eq. (94) in presence of asymmetries in the parasitic elements of the circuit. The three dark grey lines are the analytical predictions for $\delta C/C_0$ being equal to 0 (dotted), 0.05 (dashed) and 0.2 (plain). The circles, squares and diamonds are the simulated results for the values $\delta R/R$, $\delta L/L$, and $\delta C/C$ reported in the legend. We have assumed $L/L_0 = 10^{-2}$, $R/Z_{\rm out} = 10^{-1}$, $\omega_s = (2\pi)7$ GHz, $\omega_m = (2\pi)80$ MHz, $\gamma_r \simeq \gamma_t = (2\pi)0.15$ MHz, $\gamma_b = (2\pi)80$ Hz, $g_1 = (2\pi)7$ kHz, and $\bar{n}_e = \bar{n}_m = 0$. The constant photon flux $|\tilde{\alpha}|^2 = 1.15 \cdot 10^{15}$ s^{-1} has been chosen to have $n_b(t \to \infty) = 1$ for $\delta g_1/g_1 = 10^{-2}$ in the left plot.

simulations of Supplementary Eqs. (98), (99), (100), (101), and (102) ensured that asymmetries play a secondary role in the electromechanical dynamics.

As a final comment, we note that the derivation of the parameter D^2 including asymmetries can be done analytically with the procedure introduced in the Supplementary Notes 1 and 2. We do not report the result here since including δR, δL and δC makes the expressions long and complicated. In principle, however, the parameter λ for the most general antisymmetric setup can be derived analytically, with the simple expressions for λ_b and λ_p (given in the main text) being valid for small asymmetries.

SUPPLEMENTARY NOTE 3

Measurement

The parameter λ specifies how suitable a specific experimental setup is for carrying out the QND detection. The outcome of an experimental run will, however, depend on the procedure used in the experiment. In this subsection, we consider the protocol presented in the main text and illustrated in supplementary Fig. 8. We assume that the mechanical oscillator is prepared and continuously cooled to some average phonon number $\bar{n}_m$ during the entire measurement sequence. We determine the best experimental parameter Δn_b to be used to maximize the visibility ξ for a given value of λ. Finally, we compare the analytical model with numerical simulations.

First, we develop a model for the protocol presented in supplementary Fig. 8, where we only allow for a single jump during the measurement time T. After the state preparation, the membrane is in a mixture of the Fock states $\hat{\rho}_{\rm in} = \sum_i p_i |i\rangle\langle i|$, where $p_i = \bar{n}_m^i / [1 + \bar{n}_m]^{i+1}$ is the probability to be in the i–th state. When we start probing the system, the measurement outcome V_M follows a probability distribution that depends on three parameters: $\bar{n}_m$, Δn_b,

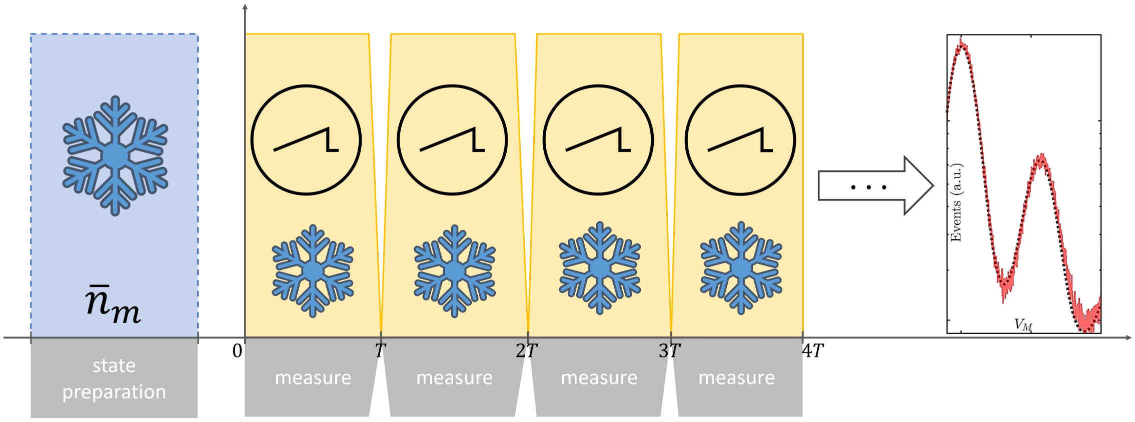

Supplementary Fig. 8: **Considered measurement sequence**. The mechanical state is cooled down to some average thermal phonon number $\bar{n}_{\mathrm{m}}$, and then measured several times, while keeping the cooling active.

and D^2. $\bar{n}_{\mathrm{m}}$ determines the initial thermal state of the membrane, and fixes the probabilities p_i. Δn_{b} determines the rate at which the membrane jumps out the ground state, and can be used to calculate the probability, for each Fock state in $\hat{\rho}_{\mathrm{in}}$, to jump up or down. In particular, the ground state $|0\rangle$ has a probability $p_0\,(1-\exp[-\Delta n_{\mathrm{b}}])$ to jump up, while any other Fock state $|i\rangle$ has probabilities $p_i\,(1-\exp[-(i+1)\Delta n_{\mathrm{b}}])$ and $p_i\left(1-\exp\left[-i\Delta n_{\mathrm{b}}(\bar{n}_{\mathrm{m}}^{-1}+1)\right]\right)$ to jump up or down, respectively. Finally, D^2 sets the distance between measurement outcomes with different phonon occupations. Therefore, whenever the membrane remains in the same state during the whole duration T, the outcome V_{M} is a Gaussian distributed random number with average $n_{\mathrm{b}}d$ and variance $\sigma^2 = d^2/D^2$. If, on the other side, the mechanical state changes during the measurement, then V_{M} is again a Gaussian distributed random number with variance σ^2, but with an average value given by $T^{-1}\left[n_{\mathrm{b}}^{(i)}T_{\mathrm{j}}d + n_{\mathrm{b}}^{(f)}(T-T_{\mathrm{j}})d\right]$. Here, T_{j} is the (uniformly distributed) random time at which the jump happens, and $n_{\mathrm{b}}^{(i)}$ and $n_{\mathrm{b}}^{(f)}$ are the phonon numbers before and after the jump. Therefore, knowing Δn_{b}, D^2 and $\bar{n}_{\mathrm{m}}$, the probability distribution function PDF of the outcomes V_{M} can be determined:

$$\mathrm{PDF}(V_{\mathrm{M}}) = \lim_{N_{\mathrm{p}}\to\infty}\left\{\sum_{i=0}^{N_{\mathrm{p}}}\frac{P_{\mathrm{p}}(i)}{\sqrt{2\pi}\sigma}e^{-\frac{(V_{\mathrm{M}}-id)^2}{2\sigma^2}} + \sum_{i=1}^{N_{\mathrm{p}}}\int_{(i-1)d}^{id}\frac{P_{\mathrm{r}}(i-1;1)}{\sqrt{2\pi}d\sigma}e^{-\frac{(V_{\mathrm{M}}-V)^2}{2\sigma^2}}dV\right\}, \tag{103}$$

where, we recall, $D = d/\sigma$, and $\sigma^2 = (\Delta V_{\mathrm{M}})^2$ is a scaling factor. In this last equation, $P_{\mathrm{p}}(i)$ is the probability for the mechanical state to remain in $|i\rangle$ for the whole measurement, while $P_{\mathrm{r}}(i-1;i)$ is the likelihood that either $|i-1\rangle$ jumps up or $|i\rangle$ jumps down. The parameter N_{p} is the size of the Hilbert space of the mechanical subsystem. Since we are interested in the peaks relative to $n_{\mathrm{b}} = 0$ and $n_{\mathrm{b}} = 1$, we can assume $N_{\mathrm{p}} = 1$ and thus rewrite (103) in the following form:

$$\begin{aligned}\mathrm{PDF}(V_{\mathrm{M}}) \simeq \sqrt{\frac{1}{2\pi\sigma^2}}&\left(\frac{e^{-\frac{V_{\mathrm{M}}^2}{2\sigma^2}-\Delta n_{\mathrm{b}}}}{1+\bar{n}_{\mathrm{m}}} + \frac{e^{-\frac{(V_{\mathrm{M}}-d)^2}{2\sigma^2}-\Delta n_{\mathrm{b}}\left(3+\bar{n}_{\mathrm{m}}^{-1}\right)}\bar{n}_{\mathrm{m}}}{(1+\bar{n}_{\mathrm{m}})^2}\right)\\ &+\left[(1+\bar{n}_{\mathrm{m}})(1-e^{-\Delta n_{\mathrm{b}}}) + \bar{n}_{\mathrm{m}}\frac{1-e^{-\Delta n_{\mathrm{b}}(3+\bar{n}_{\mathrm{m}}^{-1})}}{1+e^{-\Delta n_{\mathrm{b}}(1-\bar{n}_{\mathrm{m}}^{-1})}}\right]\frac{\mathrm{Erf}\left(\frac{V_{\mathrm{M}}}{\sqrt{2}\sigma}\right)-\mathrm{Erf}\left(\frac{V_{\mathrm{M}}-d}{\sqrt{2}\sigma}\right)}{2d(1+\bar{n}_{\mathrm{m}})^2},\end{aligned} \tag{104}$$

where $\mathrm{Erf}(\cdot)$ denotes the error function. From Supplementary Eq. (104), we can derive an analytical expression for the visibility that depends on the mentioned parameters: $\xi\left(\bar{n}_{\mathrm{m}}, D^2, \Delta n_{\mathrm{b}}\right)$. Importantly, D^2 is a dummy parameter, since $\lambda = D^2/\Delta n_{\mathrm{b}}$. It follows that, for given λ and $\bar{n}_{\mathrm{m}}$, we can maximise $\xi\left(\bar{n}_{\mathrm{m}}, \lambda\Delta n_{\mathrm{b}}, \Delta n_{\mathrm{b}}\right)$ by tuning Δn_{b}. Using this model, we obtain the analytical expression for the optimal ξ in the limit of $\lambda \gg 1$, presented in Supplementary Eq. (5) of the main text. Note that tuning Δn_{b} can be done by adjusting the probe power and choosing an appropriate measurement time T.

To investigate the validity of the single jump approximation used above, we simulate the probability distribution function for the outcome V_{M} in a Monte–Carlo simulation. The results are presented in supplementary Fig. 9. In the simulation, we allow multiple jumps to happen by dividing the measurement time T into smaller segments,

during which the mechanics is allowed to change. Similar to above, the outcome of the measurement can then be sampled from a Gaussian distribution of variance σ^2 and a mean determined by the average phonon number during the measurement. The hardly visible deviation between the analytical prediction and numerics in supplementary Fig. 9 comes from the single jump restriction, and can be eliminated by including the two–jumps events in the model. Note that, since the optimal $\Delta n_{\rm b}$ decreases for higher values of λ, the single jump approximation becomes more and more accurate with increasing λ. Secondly, for given λ and $\bar{n}_{\rm m}$, we can compare the analytical with the numerical

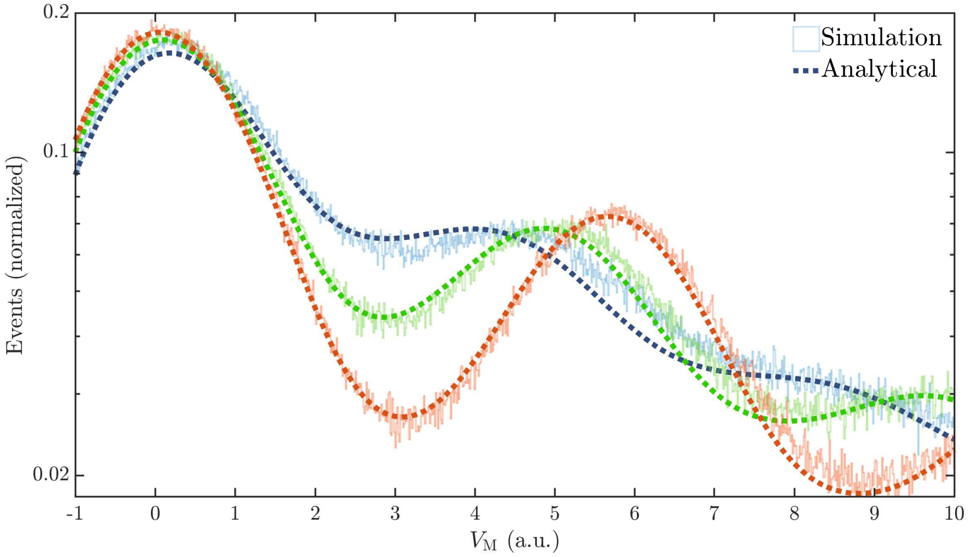

Supplementary Fig. 9: **Experimental outcomes for different** λ. Analytical (dotted curves, Supplementary Eq. (104)) and numerical (solid, noisy curves) probability distribution function for the experimental outcome $V_{\rm M}$. We choose $\bar{n}_{\rm m} = 1$ and λ equal to 20 (blue), 100 (green) and 200 (red). The values of $\Delta n_{\rm b}$ that have been used were determined using the analytical maximization of the visibility ξ, with the cited λ and $\bar{n}_{\rm m}$ as free parameters.

maximum visibility ξ. The latter is determined using repeated Monte–Carlo simulations with different values for $\Delta n_{\rm b}$, as shown in supplementary Fig. 10. Each point in such plot corresponds to a single simulation with given parameters λ, $\bar{n}_{\rm m}$ and $\Delta n_{\rm b}$. The error bars are derived assuming Poissonian statistics in each bin of the histogram collecting the outcomes $V_{\rm M}$. A polynomial fit is then used for determining the maximum visibility ξ, that is compared with the analytical prediction in supplementary Fig. 3 of the main text (2D plot; analytical corresponds to the red dotted line, numerical results are represented by blue circles).

As a final remark, it is important to say that the results of this section can easily be adapted to other experimental schemes [5]. For instance, it is possible to first cool down the mechanical motion to the ground state, and then let it thermalize while measuring several times. An advantage of this approach is that it reduces the probability of jumping down from the excited Fock states, since the cooling is absent. On the other hand, operating the experiment in a pulsed regime may add an extra degree of complexity, e.g., transient effects associated with the change in equilibrium position of the membrane when the fields are turned on.

SUPPLEMENTARY NOTE 4

Realistic experimental parameters

Here we study in more detail a possible experimental setup for our proposal, and the potential challenges that may arise pursuing the QND measurement of the phonon number. First, we present a simple derivation for determining the linear and quadratic electromechanical couplings g_1 and g_2 [9]. Then, we discuss the presence of a stray capacitance $C_{\rm s}$, the main effect of which is to reduce these couplings. Finally, we analyse the feasibility of an experiment, considering aspects such as the intracavity photon number, the mechanical quality factor, the measurement time, and probe power. The parameters employed are the same as introduced in the main text: $\omega_{\rm s} = (2\pi)7$ GHz, $\omega_{\rm m} = (2\pi)80$ MHz, $\gamma_{\rm r} \simeq \gamma_{\rm t} = (2\pi)150$ kHz, $R = Z_{\rm out}/10$, $\bar{n}_{\rm e} = 0$, $g_{\rm r} = 10^{-2} g_1$ and $C_{\rm s} = 100 C_0$. The membrane is assumed to be 1 μm long and 0.3 μm wide, with a quality factor $Q = 10^6$ (for graphene membranes at cryogenic temperatures, quality

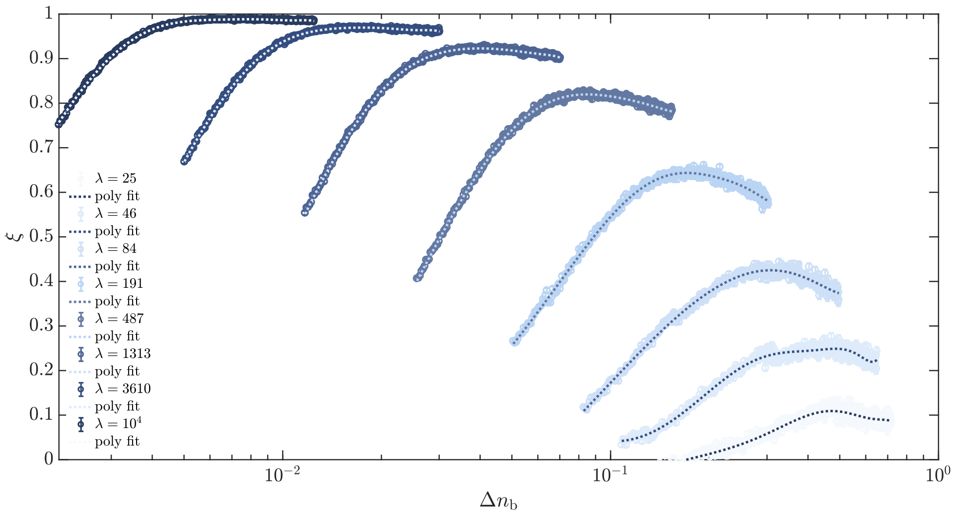

Supplementary Fig. 10: **Numerical optimization of the visibility**. Visibility as a function of the parameter Δn_{b}, for different values of λ. The points are derived numerically using the Monte–Carlo method, and the dotted lines are polynomial fit. Notice that error bars are included, and have been determined assuming Poissonian statistics in each bin of the histogram collecting the outcomes V_{m}.

factors between 10^5 and 10^6 have been showed in [10]). We discuss the average occupation $\bar{n}_{\mathrm{m}}$ of the mechanical bath in the following.

The mechanical membrane is fixed along all its boundaries, such as in supplementary Fig. 1(a) of the main text. A basis of modes describing its motion is thus $\{u_{i,j}(x,y)\}$, where

$$u_{i,j}(x,y) = \epsilon \sin\left(i\frac{\pi}{L}x\right)\sin\left(j\frac{\pi}{W}y\right). \tag{105}$$

Here, L and W are the length and width of the membrane, respectively. The mode of interest has indices $i = 2$ and $j = 1$, and the constant ϵ fixes an effective gauge for the mass. By setting $\int_0^L \int_0^W |u_{i,j}(x,y)|^2\, dxdy = 1$, we choose the gauge in which the so–called effective mass is the physical mass, and $\epsilon = 2$. Recalling that

$$g_1 = \frac{x_0 \omega_{\mathrm{s}}}{C_0} \frac{\partial C}{\partial \beta}\Big|_{x=0}, \tag{106}$$

$$g_2 = \frac{x_0^2 \omega_{\mathrm{s}}}{2C_0} \frac{\partial^2 C}{\partial \beta^2}\Big|_{x=0}, \tag{107}$$

the couplings can be determined once the derivatives are found. As discussed in Ref. [9], β describes the amplitude of the considered mode, and can be viewed as a canonical position. An approximate value of the derivatives in Supplementary Eq. (106) and (107) is then

$$\frac{\partial^k C}{\partial \beta^k}\Big|_{x=0} \simeq (-1)^k k! \frac{C_0}{d^k} \frac{2}{LW} \int_0^{L/2} \int_0^W u_{2,1}^k(x,y) dxdy, \tag{108}$$

where d is the distance separating the two capacitor's plates, and the integral is taken over half the membrane, for the reasons discussed in the Supplementary Note 2. Using Supplementary Eqs. (106), (107) and (108), we can finally determine the values of g_1 and g_2 to be

$$g_1 = \frac{8}{\pi^2} \frac{x_0 \omega_{\mathrm{s}}}{d}, \tag{109}$$

$$g_2 = \frac{x_0^2 \omega_{\mathrm{s}}}{d^2}. \tag{110}$$

For the parameters introduced in the main text, we find $g_1 \simeq (2\pi)715$ kHz and $g_2 \simeq (2\pi)111$ Hz. Considering that we assumed an electrical damping $\gamma_{\rm t} = (2\pi)150$ kHz, it could seem that we reach the strong coupling regime $g_1 > \gamma_{\rm t}$. However, we have so far ignored stray capacitances, that are the main reason for which the strong coupling regime (and thus the phonon QND measurement) has never been accomplished in electromechanics. For such small geometries, the stray capacitance $C_{\rm s}$ always exceed the intrinsic capacitance C_0 by up to several orders of magnitude [10], and severely limits the attainable values of the linear and quadratic couplings [see supplementary Fig. 11(b)]. Looking at Supplementary Eqs. (106), (107), and considering that the stray capacitance does not affect the derivatives $\partial^k C/\partial\beta^k$, we replace the coupling constants by

$$g_1 \to \frac{C_0}{C_0 + C_{\rm s}} g_1, \tag{111a}$$

$$g_2 \to \frac{C_0}{C_0 + C_{\rm s}} g_2. \tag{111b}$$

For $C_{\rm s} = 100C_0$, we find $g_1 \simeq (2\pi)7$ kHz and $g_2 \simeq (2\pi)1$ Hz. This value of $C_{\rm s}$ is optimistic for geometries similar to the ones in Ref. [10, 11], where graphene sheets are laid on a substrate in such a way that the contact area between the two interfaces is very large. However, for a setup like the one described in Ref. [12], membranes of similar dimensions as the ones conjectured here are assembled onto small localized gates, dramatically reducing the stray capacitance. With this fabrication technique, a stray capacitance of 50 fF is obtained, for a membrane that is about two and a half times the size of the one conjectured here. In our case, this value for the stray capacitance would correspond to 376 times C_0, where $C_0 \simeq 0.13$ fF. Due to the smaller size of our membrane, we assume a reduction in the stray capacitance, such that we consider $C_{\rm s} = 100C_0$.

In our settings, reaching the strong coupling would require $C_{\rm s} < 3.8C_0$. In this regime, phonon QND detection could also be performed with the strategy proposed in [7]. With a large stray capacitance, however, we cannot accomplish such requirement, and investigate different approaches to QND detection. With the experimental parameter described above, we find $\lambda_{\rm b} = 105 \times Z_{\rm out}/R$ and $\lambda_{\rm p} = 0.014(g_1/g_{\rm r})^2$, regardless of the value of $C_{\rm s}$. The highest quoted value $\lambda = 122$ is found assuming $g_{\rm r} = 10^{-2} g_1$ and $Z_{\rm out} = 10R$.

In the remainder of this section, we study the conditions under which the QND detection could be implemented, focusing on the incident power and the measurement time. First, we recall that the membrane's heating has several contributions. Two of them, denoted $\Delta n_{\rm b}^{(b)}$ and $\Delta n_{\rm b}^{(p)}$, are the ones identified in Supplementary Eqs. (89) (rhs, second term) and (93), respectively. They describe the feedback of the electrical system on the mechanical motion, and are the denominators of the parameters $\lambda_{\rm b}$ and $\lambda_{\rm p}$ defined in the main text. The third contribution comes from the mechanical reservoir. Indicated with $\Delta n_{\rm b}^{(m)}$, this is given in Supplementary Eq. (89) (rhs, first term), and is independent of the strength of the probing field. For determining the parameter λ, we have so far ignored $\Delta n_{\rm b}^{(m)}$, assuming that the measurement is fast enough. This is an excellent approximation, as far as $\Delta n_{\rm b}^{(m)} \ll \Delta n_{\rm b}^{(b)} + \Delta n_{\rm b}^{(p)}$. Below, we discuss the case in which there is a seizable contribution from the mechanical bath, and describe its effect on the QND detection of the phonon number.

Above we have seen that, for the considered experimental parameters, $\lambda = 122$, for which we find the optimal $\Delta n_{\rm b} = 0.21$. For $C_{\rm s} = 100C_0$, the total number of photons that we need to send within the measurement is $\alpha^2 \simeq 4.5 \cdot 10^{11}$ [see Supplementary Eqs. (89) and (93)]. By sending these photons within a sufficiently short time, we can neglect the influence of the mechanical reservoir. To derive precise conditions for this, we define the effective temperature $\bar{N}_{\rm e}$ of the electrically induced reservoir:

$$\bar{N}_{\rm e} = \frac{\Delta n_{\rm b}^{(b)} + \Delta n_{\rm b}^{(p)}}{T(\gamma_{\rm b} + \Gamma_{\rm b})}. \tag{112}$$

This last equation is derived from Supplementary Eq. (94), by sending the time t to infinity, and recalling the definitions of $\Delta n_{\rm b}^{(b)}$ and $\Delta n_{\rm b}^{(p)}$. $\bar{N}_{\rm e}$ is the average phonon number at which the membrane stabilizes in the absence of the mechanical reservoir. The condition $\Delta n_{\rm b}^{(m)} \ll \Delta n_{\rm b}^{(b)} + \Delta n_{\rm b}^{(p)}$, under which the mechanical bath can be neglected, can be rewritten as $\bar{n}_{\rm m} \ll \bar{N}_{\rm e}$. The measurement time T is thus a knob that allows us to adjust the relative weights of the mechanical reservoir and the electrical feedback on the membrane. For short measurement times, $\bar{N}_{\rm e}$ is increased, and this condition is easier to satisfy. As an example, if we choose $T = 0.4$ ms and $Q = 10^6$, we get $\bar{N}_{\rm e} = 1$, implying that the average occupation of the membrane's reservoir needs to be less than unity to neglect the mechanical reservoir. For our setup, this requires the temperature of the cryostat to be lower than 6 mK, a challenging task for current technology. On the other side, choosing $T = 0.04$ ms fixes $\bar{N}_{\rm e} = 10$, and the temperature for which $\bar{n}_{\rm m}$ becomes negligible is 40 mK, that has been already achieved in experiments involving graphene resonators [10]. Once the measurement time is chosen, and assuming the photon flux $|\tilde{\alpha}|^2 = |\alpha|^2/T$ to be constant, one can determine the

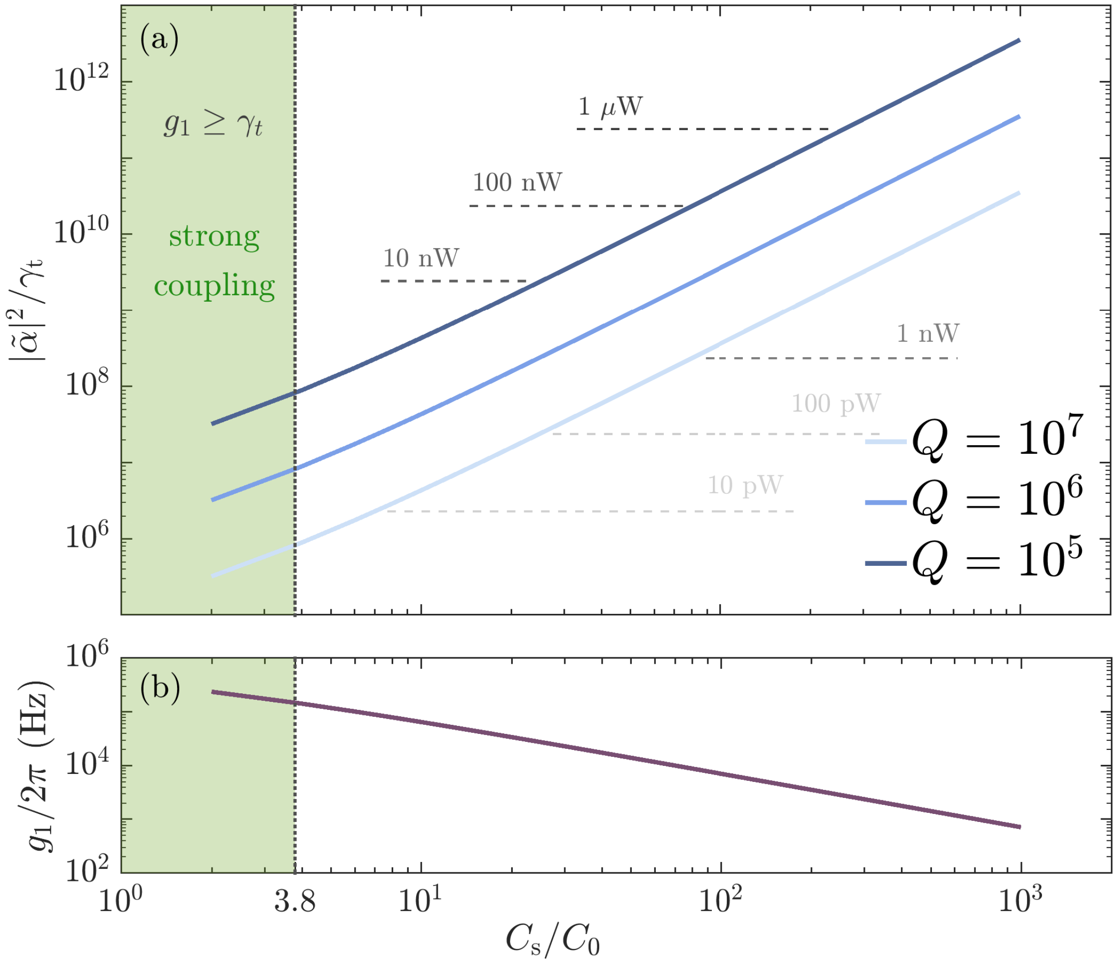

Supplementary Fig. 11: **Operating conditions in the presence of stray capacitances**. **(a)** Average intracavity photons $\tilde{\alpha}^2/\gamma_t$ as a function of the (normalized) stray capacitance. The three lines correspond to different values of the mechanical quality factor, as indicated in the legend. We assume $\bar{N}_e = 3$, and equal heating contribution from the mechanical and electrically induced reservoirs, with $\bar{n}_m = 3$. **(b)** Linear coupling g_1 as a function of the relative value of the stray capacitance C_s/C_0. For both figures, in the shadowed region the strong coupling $g_1 \geq \gamma_t$ is achieved.

probing power P_{in} and the average intracavity photon number $|\tilde{\alpha}|^2/\gamma_t$. As an example, with $T = 0.4$ ms, P_{in} becomes 5.3 nW and $|\tilde{\alpha}|^2/\gamma_t = 1.2 \cdot 10^9$. These parameters are highly dependent on the mechanical quality factor Q, and the stray capacitance, as investigated below in supplementary Fig. 11(a).

Finally, we discuss the case in which the electrical heating is in equilibrium with the heating and damping of the mechanical reservoir. In that case, we need to renormalize λ by the total heating:

$$\lambda' = \frac{\Delta n_b^{(b)} + \Delta n_b^{(p)}}{\Delta n_b^{(m)} + \Delta n_b^{(b)} + \Delta n_b^{(p)}} \lambda, \tag{113}$$

where λ contains both the terms λ_b and λ_p.

Importantly, we can always make $\lambda' \simeq \lambda$ by increasing the driving strength and reducing the measurement time (thus decreasing $\Delta n_b^{(m)} = \bar{n}_m \gamma_b T$). The parameter λ' substitutes λ in the description of the system. The mechanical bath thus reduces the quality of the phonon QND measurement, but the analysis above still applies, with λ' instead of λ. As an example, assume that the temperature is 14 mK [10]. Then, the average occupation of the membrane's reservoir is $\bar{n}_m = 3$. We choose T such that the electrical heating is equal to the mechanical: $\bar{N}_e = \bar{n}_m$. Then

$\lambda' = \lambda/2 = 61$ with the previous parameters. This value is well in the regime of good visibility ξ, and for this λ', the optimal value of the total heating is $\Delta n_{\rm b} = \Delta n_{\rm b}^{(m)} + \Delta n_{\rm b}^{(b)} + \Delta n_{\rm b}^{(p)} = 0.3$. Depending on the value of the mechanical quality factor Q and stray capacitance $C_{\rm s}$, we can then determine the required incident power and the intracavity photon number, as shown in supplementary Fig. 11. Since we assume $\Delta n_{\rm b}^{(m)} = \Delta n_{\rm b}/2$, the measurement time is $T = \Delta n_{\rm b}/(2\gamma_{\rm b}\bar{n}_{\rm m}) \simeq 0.05Q/\omega_{\rm m}$. For the parameters in the figure and a quality factor Q varying between 10^5 and 10^7, we find $T \in \left[10^{-5}, 10^{-3}\right]$ s.

SUPPLEMENTARY REFERENCES

[1] Devoret, M. H. *et al.* Quantum fluctuations in electrical circuits. *Les Houches, Session LXIII* **7** (1995).

[2] Clerk, A. A., Devoret, M. H., Girvin, S. M., Marquardt, F. & Schoelkopf, R. J. Introduction to quantum noise, measurement, and amplification. *Reviews of Modern Physics* **82**, 1155 (2010).

[3] He, B., Yang, L., Lin, Q. & Xiao, M. Radiation pressure cooling as a quantum dynamical process. *Phys. Rev. Lett.* **118**, 233604 (2017). URL `https://link.aps.org/doi/10.1103/PhysRevLett.118.233604`.

[4] Bagci, T. *et al.* Optical detection of radio waves through a nanomechanical transducer. *Nature* **507**, 81–85 (2014).

[5] Dellantonio, L., Kyriienko, O., Marquardt, F. & Sørensen, A. In preparation.

[6] Miao, H., Danilishin, S., Corbitt, T. & Chen, Y. Standard quantum limit for probing mechanical energy quantization. *Phys. Rev. Lett.* **103**, 100402 (2009). URL `https://link.aps.org/doi/10.1103/PhysRevLett.103.100402`.

[7] Thompson, J. D. *et al.* Strong dispersive coupling of a high-finesse cavity to a micromechanical membrane. *Nature* **452** (2008). URL `http://www.nature.com/nature/journal/v452/n7183/suppinfo/nature06715_S1.html`.

[8] Dalibard, J., Castin, Y. & Mølmer, K. Wave-function approach to dissipative processes in quantum optics. *Physical review letters* **68**, 580 (1992).

[9] Zeuthen, E. *Electro-Optomechanical Transduction & Quantum Hard-Sphere Model for Dissipative Rydberg-EIT Media*. Ph.D. thesis, The Niels Bohr Institute, Faculty of Science, University of Copenhagen (2015).

[10] Singh, V., Bosman, J., S., Schneider, H., B., Blanter, M., Y. & Castellanos-Gomez, G. A., A. Steele. Optomechanical coupling between a multilayer graphene mechanical resonator and a superconducting microwave cavity. *Nat Nano* **9**, 820–824 (2014).

[11] Weber, P., Güttinger, J., Tsioutsios, I., Chang, D. E. & Bachtold, A. Coupling graphene mechanical resonators to superconducting microwave cavities. *Nano Letters* **14**, 2854–2860 (2014). URL `http://dx.doi.org/10.1021/nl500879k`. PMID: 24745803, http://dx.doi.org/10.1021/nl500879k.

[12] Song, X. *et al.* Stamp transferred suspended graphene mechanical resonators for radio frequency electrical readout. *Nano Letters* **12**, 198–202 (2012). URL `http://dx.doi.org/10.1021/nl203305q`. PMID: 22141577, http://dx.doi.org/10.1021/nl203305q.
